# Inhibition of neurite outgrowth and enhanced effects compared to baseline toxicity in SH-SY5Y cells

**DOI:** 10.1007/s00204-022-03237-x

**Published:** 2022-02-19

**Authors:** Jungeun Lee, Beate I. Escher, Stefan Scholz, Rita Schlichting

**Affiliations:** 1 Department of Cell Toxicology, Helmholtz Centre for Environmental Research-UFZ, Leipzig, Germany; 2 Environmental Toxicology, Center for Applied Geoscience, Eberhard Karls University Tübingen, Tübingen, Germany; 3 Department of Bioanalytical Toxicology, Helmholtz Centre for Environmental Research-UFZ, Leipzig, Germany

**Keywords:** Developmental neurotoxicity, Neurite outgrowth, Specificity, Enhanced toxicity, Pesticides

## Abstract

Early life exposure to environmental chemicals can cause developmental neurotoxicity (DNT). The impairment of key neurodevelopmental processes such as neurite outgrowth inhibition can be used as endpoints for screening of DNT effects. We quantified neurite-specific effects using the ratio of effect concentrations for cytotoxicity and neurite outgrowth inhibition (SR_cytotoxicity_). Baseline cytotoxicity, the minimal toxicity of any chemical, was used to quantify enhanced cytotoxicity (toxic ratio, TR) and neuronal-specific toxicity (SR_baseline_) by comparing baseline cytotoxicity with the effects on cell viability and neurite outgrowth, respectively. The effects on cell viability and neurite length were measured based on image analysis in human neuroblastoma SH-SY5Y cells. Baseline cytotoxicity was predicted from hydrophobicity descriptors using a previously published model for SH-SY5Y cells. Enhanced cytotoxicity and neuronal-specific toxicity were more often observed for hydrophilic chemicals, which indicates that they are more likely to act through specific modes of action (MOA) on cell viability and neurite outgrowth. Hydrophobic chemicals showed a tendency to act through baseline toxicity without showing specific or enhanced toxicity, but were highly potent considering their low effect concentrations for both cytotoxicity and neurite outgrowth inhibition. The endpoint-specific controls (narciclasine, colchicine, cycloheximide, and rotenone), two carbamates (3-hydroxycarbofuran and carbaryl), and two redox cyclers (diquat and paraquat) showed distinct neurite-specific effects (SR_cytotoxicity_ > 4). By comparing neurite-specific effects with enhanced cytotoxicity, one can explain whether the observed effects involve specific inhibition of neurite outgrowth, other specific MOAs, or merely baseline toxicity arising from hydrophobicity.

**Supplementary Information:**

The online version contains supplementary material available at 10.1007/s00204-022-03237-x.

## Introduction

The developing nervous system is vulnerable to exposure to environmental chemicals (Giordano and Costa 2012). Despite the high relevance for human health, developmental neurotoxicity (DNT) is only conditionally considered in chemical safety assessment by current OECD test guideline (OECD TG 426). These test guidelines represent in vivo test with developing rats and are very demanding in terms of animal numbers and labor. Therefore, in vitro bioassays may serve as animal-protective and time- and resource-efficient alternatives to animal testing and enable high-throughput screening of environmental chemicals for routine assessment of DNT. Due to the diversity in molecular initiating events (MIE) leading to DNT and limited mechanistic information of the cellular toxicity pathways leading to DNT, the key neurodevelopmental processes are considered as endpoints for testing DNT in vitro rather than the assessment of MIEs (Bal-Price et al. 2015; Lein et al. 2005; Smirnova et al. 2014). Neurite outgrowth, in particular, is an important step in the differentiation of the nervous system as the basis for connectivity and function of neural network in the nervous system, and diverse in vitro models are available to assess effects on neurite outgrowth (Masjosthusmann et al. 2020; Radio and Mundy 2008).

DNT has been reported for many environmental chemicals, in particular, pesticides (Bjorling-Poulsen et al. 2008; Grandjean and Landrigan 2006). Pesticides control pests through diverse mechanisms and many insecticides target specific sites in nervous system such as acetylcholinesterase (AChE), acetylcholine receptor (AChR), voltage-gated sodium channel, and γ-aminobutyric acid (GABA) receptor (Casida 2009; Lushchak et al. 2018). DNT of pesticides in non-target organisms is supported by experimental and epidemiological evidence (Bjorling-Poulsen et al. 2008), and commonly used pesticides were confirmed to inhibit neurite outgrowth in PC-12 cells (Christen et al. 2017). Especially, many pesticides of concern for DNT in humans or animals provoked effects in multiple DNT-related endpoints in DNT in vitro testing battery (Masjosthusmann et al. 2020).

In vitro tools have been applied to screen toxicants causing DNT and capture the specific effects on neurite outgrowth. The U.S. National Toxicology Program (NTP) provided a proof-of-concept chemical library (Behl et al. 2019) for testing neurotoxicity and DNT, and high-throughput screening for neurite outgrowth inhibition has been performed on these NTP library compounds (Delp et al. 2018; Ryan et al. 2016). These screening studies quantified specificity of the DNT effects by comparing the ratio between effect concentrations or benchmark concentrations derived for neurite outgrowth inhibition and cytotoxicity, and demonstrated that specific effects on neurite outgrowth inhibition can be distinguished from general cytotoxic effects. Masjosthusmann et al. (2020) applied multiple DNT assays to chemicals presumed to be developmental neurotoxicants and negative controls, and the endpoints related to neurite morphology were the most sensitive in 11% of 119 chemicals if the neuronal network formation assay was excluded and 7% of 60 chemicals if it was included (Masjosthusmann et al. 2020). While specificity of DNT was evaluated by comparing the effect concentrations to levels of cytotoxicity in these studies, the distance to levels of baseline toxicity, which is the minimal toxicity of any chemical, can provide further understanding of the observed DNT effects.

Baseline toxicity represents a nonspecific mode of action (MOA) and results from membrane interference of chemicals. Baseline toxicity is driven by hydrophobicity of chemicals, and can be assessed and predicted easily in experimental systems with a partition-based exposure, but is applicable to any organism and cell type. The interference of chemicals with membranes leads to a critical membrane burden causing 10% cytotoxicity that was reported to stay constant (69 mmol^.^L_lip_^−1^) over diverse mammalian cells (Escher et al. 2019). Accordingly, a quantitative structure–activity relationship (QSAR) was developed to predict nominal concentration of baseline cytotoxicity for multiple in vitro assays including also human neuroblastoma SH-SY5Y cells (Lee et al. 2021). Hydrophobicity—described by partition constants between liposomes (membrane bilayer vesicles) and water (*K*_lip/w_)—serves as a single descriptor of the baseline cytotoxicity QSAR (Lee et al. 2021).

The predicted baseline cytotoxicity has been applied to estimate how potent the observed toxicity is for the target endpoint compared to the minimal toxicity, and the enhanced toxicity over baseline cytotoxicity indicates the involvement of specific MOA (Escher et al. 2020). The current approach using cytotoxicity as a reference for DNT is useful to quantify how important neurite outgrowth is compared to general cytotoxic effects on neuronal cells that integrate all modes of action leading to cytotoxicity. In contrast, baseline toxicity is independent of cell type (or organism), and, therefore, can provide additional metrics to quantify any elevated toxicity that occurs in neuronal cells compared to nonspecific effects from baseline toxicity. Many pesticides are highly hydrophobic, and hence, they already provoke strong toxic effects via baseline toxicity. However, it has not been explored yet if pesticides exert specific MOA leading to enhanced cytotoxicity or toxicity to the target endpoint compared to baseline cytotoxicity in the neuronal cells. Therefore, these additional measures considering baseline toxicity can provide further details to the current approach considering the ratio of effects on neurite outgrowth to cytotoxicity.

We aim to identify the degree of specificity and elevated cytotoxicity of effects for pesticides and environmental chemicals on neurite outgrowth. Differentiated SH-SY5Y cells were used to test the effects of chemicals on cell viability and neurite outgrowth. Effect concentrations were then compared to predicted baseline cytotoxicity using QSAR developed for differentiated SH-SY5Y cells (Lee et al. 2021). SH-SY5Y cells can be differentiated into more mature neuron-like cells, and retinoic acid is commonly applied for differentiation (Agholme et al. 2010; Biedler et al. 1973; Kovalevich and Langford 2013; Påhlman et al. 1984). The cell viability and neurite length were measured by image analysis. The focus was set on pesticides that target nervous system or energy metabolism. For comparison, we included the assessment of endpoint-specific controls, i.e., highly specific positive controls for neurite outgrowth (Aschner et al. 2017; Krug et al. 2013), including narciclasine, colchicine, cycloheximide, and rotenone, all of which are natural plant-derived chemicals. Confirmed baseline toxicants (Vaes et al. 1998) were applied as negative controls. Additional chemicals from the NTP (US National Toxicology Program) library such as endocrine disrupting chemicals were also tested for comparison. The test chemicals were then classified based on their specific effects on neurite outgrowth.

## Materials and methods

### Chemicals

Endpoint-specific positive controls for neurite outgrowth (Aschner et al. 2017), known baseline toxicants (Vaes et al. 1998), and pesticides with diverse MOAs (Casida 2009) and some endocrine disrupting chemicals (EDCs) were tested in this study (Table S1). Additionally, polycyclic aromatic hydrocarbons (PAHs), polybrominated diphenyl ethers (PBDEs), and polychlorinated biphenyls (PCBs) from the NTP library were tested for comparison (Table S2). The chemical stocks were prepared in methanol. For chemicals with higher water solubility, methanol was evaporated under a stream of nitrogen gas prior to adding the appropriate amount of assay medium. For chemicals with low solubility, the stock solution was directly added to dosing medium and the final concentration of methanol in assay plates was limited to a maximum of 1% which was found to not cause any effects on cell viability and neurite outgrowth inhibition.

### Selection of cell model and cell culture

SH-SY5Y cells and Lund human mesencephalic (LUHMES) cells were considered as candidates for developing high-throughput screening assay detecting effects on neurite outgrowth. LUHMES cells are currently used to test effects of chemicals on neurite outgrowth in DNT in vitro battery mainly and they have the advantage of a non-oncogenic origin (Masjosthusmann et al. 2020). In the present study, we selected SH-SY5Y cells for screening effects on neurite outgrowth because of their easier maintenance and availability of commercial 384-well plates with appropriate coating for adherence of cell monolayers.

SH-SY5Y cells (Sigma-Aldrich, 94,030,304) were cultured at 37 °C in 5% CO2 in incubator. Growth medium consisted of 90% of DMEM/F12 (Gibco, 11,320,074) and 10% of heat-inactivated fetal bovine serum (Gibco, 10,500,064) with 100 U/mL penicillin and 100 µg/mL streptomycin (Gibco, 15,140,122). Cells were used from passage 5 only up to passage 15 to avoid senescence.

### Plating cells and dosing

Before the assay, SH-SY5Y cells were differentiated in flasks for 72 h using 10 µM all-trans retinoic acid (Sigma-Aldrich, R2625). The differentiation medium was composed of Neurobasal™ medium with phenol-red (Gibco, 21,103,049) supplemented with 2% B-27™ Supplement (Gibco, 17,504,044), 2 mM GlutaMAX™ (Gibco, 35,050,061), and 100 U/mL penicillin and 100 µg/mL streptomycin. For seeding and dosing, phenol-red free Neurobasal™ medium (Gibco, 12,348,017) was used as differentiation medium.

The differentiated cells were plated at density of 3,000 cells/well in Collagen I-coated 384-well plates (Corning, 354,667). 30 µL medium containing differentiated cells and 10 µM all-trans retinoic acid were added into each well using a MultiFlo™ Dispenser (Biotek, Vermont, USA). The last two columns of each plate were used as control with or without cells. The seeded cells were incubated for further 24 h in the incubator.

Dosing medium was prepared either by directly adding chemical stocks or blowing down stock solution with nitrogen gas. The dosing medium was then diluted in serial or linear dilution, and 10 µL of diluted dosing medium was transferred to the plates using a pipetting robot (Hamilton Star, Bonaduz, Switzerland). Eleven concentrations were tested with two technical replicates for each chemical, and exposure concentrations were selected based on predicted baseline toxicity and adjusted in case limited solubility was observed. We allowed turbidity only up to the level it started to be observed by eyes and these chemicals with turbidity issue are flagged. In each assay plate, narciclasine and MeOH were included as positive control (Aschner et al. 2017; Delp et al. 2019) and solvent control, respectively. The tests were repeated at least in three independent experimental runs for the chemicals which showed effects on the first test set. The inactive chemicals were not tested further, but the predicted baseline cytotoxicity values are noted in Table S2. After dosing, the cells were kept in the incubator for 24 h.

### Neurite outgrowth measurement

Neurite length was measured and analyzed in phase-contrast image by an IncuCyte^®^ S3 live cell imaging system (Essen BioScience, Ann Arbor, Michigan, USA). After 24 h exposure, phase-contrast images were recorded in each well with a 10X objective lens, which imaged 36% of the well area. The total neurite length per image was quantified by IncuCyte^®^ NeuroTrack software module (Fig. S1), and the neurite length relative to control was used to express the effects on neurite outgrowth. The cells got clustered or partially detached in the wells where most cells were dead; therefore, the neurite length was not normalized by the cell numbers to avoid possible artifacts. In case significant stimulating effects were observed in neurite outgrowth, total neurite length divided by total cell counts was also evaluated for comparison to exclude artifacts from different cell numbers and verify the stimulating effects observed in original data analysis.

For quality assurance, phase-contrast images were taken from each well at 30 min after seeding to quantify artifacts caused by scratches on the plate bottom or fine dust fluff. When this background signal was higher than three times the standard deviation of the mean background signal, the image was flagged and checked if any artifacts were observed.

### Viability test

After capturing phase-contrast images, Nuclear Green™ LCS1 (Abcam, ab138904) and propidium iodide (Sigma-Aldrich, 81,845) were used to stain total and dead cells, respectively. The stains were diluted in phosphate-buffered saline (PBS) to make the final concentration of 10 µM Nuclear Green™ LCS1 and 1 µM propidium iodide. 10 µL of the mixture was added into each well with a multi-channel pipette and the plates were incubated for 1 h in the incubator. Fluorescence images were derived with a 10× objective lens in green (excitation wavelength: 460 nm; emission wavelength: 524 nm; acquisition time: 300 ms) and red (585 nm; 635 nm; 400 ms) fluorescence channel. The stained cell objects were counted with Basic analyzer mode in IncuCyte^®^ S3 software (Fig. S1), and cell viability was calculated by dividing the number of live cells (total-dead cells) by those of total cells. The decrease in cell viability compared to unexposed cells was defined as cytotoxicity.

### Data evaluation

The analysis model for the concentration–response curves (CRC) was selected among three models: a linear regression model, a log-logistic model, and the Brain–Cousens model (Brain and Cousens 1989; Ritz et al. 2015).

CRC typically shows linearity up to 30% effect level and the effect concentration can be derived from the slope of interpolation line as described previously by Escher et al. (2018) using Eq. (1).

1 $$\% {\text{ cell viability or neurite length}} = 100\% - {\text{slope}} \times {\text{concentration }}({\text{M}}),$$

Data up to 30% effect level were included in linear CRC analysis when no plateau was observed. The concentration leading to 10% cytotoxicity (IC_10_) and 10% neurite outgrowth inhibition (EC_10_) was determined using Eqs. 1 and 2

2 $${\text{IC}}_{{{10}}} \,{ = }\,\frac{{{\text{10}}\%}}{{{\text{slope}}}},$$

3 $${\text{EC}}_{{10 }} { = }\frac{{{\text{10}}\%}}{{{\text{slope}}}}.$$

For the log-logistic model (Eq. 4), data of all effect levels were included for analysis and the IC_10_ or EC_10_ were derived with the following equations:

4 $$\% {\text{ cell}} {\text{ viability}} {\text{ or}} {\text{ neurite}} {\text{ length}} = 100\% - \frac{100}{{1 + 10^{{\left( {\log \left( {\frac{{EC_{50} }}{{{\text{concentration}}({\text{M}})}}} \right) \times {\text{ slope}}} \right)}} }},$$

5 $$\log {\text{EC}}_{50 }= \log {\text{EC}}_{10} - \left( {\frac{1}{{{\text{slope}}}}} \right) \times \log \left( {\frac{90}{{10}}} \right).$$

Equations 1 and 4 were fitted with GraphPad prism (version 9, San Diego, California, USA). Standard errors were calculated with error propagation according to Escher et al. (2018).

The Brain–Cousens model is for hormetic *U*-shaped curves and was also applied to whole data using the drc package in *R* studio version 4.0.4 (Brain and Cousens 1989; Ritz et al. 2015). The equation that used for Brain–Cousens model is

6 $$\% {\text{ neurite length}} = c + \frac{{d - c + f \times {\text{concentration}}(\mu {\text{M}})}}{{1 + \exp (b\left( {\log ({\text{concentration}}(\mu {\text{M}})/e)} \right)}},$$

where the concentration is given in micromolar units (μM), and *b*, *c*, *d*, *f*, and *e* are adjustable parameters. The parameter *f* quantifies the degree of hormesis, that is, stimulating effects and a higher *f* implies stronger hormetic effect. The derived best-fit values of model parameters were used as input parameters to calculate EC_10_ for stimulating effects (i.e., 110% of controls) and inhibiting effects (90% of controls). EC_10_ for inhibiting effects were calculated using the ED command in R

The CRC models used to estimate effect concentrations for cell viability and neurite length were selected based on a decision tree as indicated in Fig. S2. Among the three models mentioned above, the linear regression model (Eq. 1) was applied preferentially to fit CRCs of both endpoints. When the IC_10_ and EC_10_ could not be derived with 95% confidence interval from the interpolation line of linear regression or when the data did not follow linearity (e.g., reached a plateau), a log-logistic model (Eq. 4) was applied instead. In case of neurite length, the Brain–Cousens model was applied for chemicals that stimulated neurite outgrowth. When neurite length over 110% was observed in more than two independent experimental sets, the significance of the hormesis parameter *f* was checked in Brain–Cousens model and the model was applied only when the parameter was significant (*p* value < 0.05).

### Prediction of IC_10,baseline_ from a baseline cytotoxicity QSAR for SH-SY5Y cells

Nominal concentrations for baseline cytotoxicity leading to 10% cytotoxicity (IC_10,baseline_) were predicted with a baseline toxicity prediction model based on a quantitative structure–activity relationship (QSAR) derived specifically for differentiated SH-SY5Y cells (Lee et al. 2021). IC_10_ values reported here were already published and used for application of this baseline cytotoxicity QSAR by Lee et al. (2021). The baseline toxicity prediction model can predict IC_10,baseline_ solely from the liposome–water partition constants (*K*_lip/w_) and more details of the baseline toxicity prediction model are given in Text S1. The pH-corrected liposome–water distribution ratios (*D*_lip/w_) were used for charged chemicals according to Lee et al. (2021).

### Calculation of toxic ratio and specificity ratios

The toxic ratio (TR) is a measure to estimate if the cytotoxic effects of tested chemicals are caused by a specific MOA (Maeder et al. 2004). TRs are obtained by comparing the observed cytotoxic effects (experimental IC_10_) and predicted cytotoxicity caused by baseline toxicity (IC_10,baseline_), as shown in Table 1, using the equation

7 $${\text{TR = }}\frac{{{\text{IC}}_{{\text{10,baseline}}} }}{{{\text{IC}}_{{{10}}} }}.$$

Chemicals with 0.1 < TR < 10 are typically classified as baseline toxicants, and a specific MOA is suggested for cytotoxic effects when TR > 10 (Maeder et al. 2004)

**Table 1:** Terminology for evaluation of effects in in vitro assays in general and for developmental neurotoxicity (DNT)

Description	General definition	Reference	Definition for DNT	Reference
Toxic ratio TR: specific mode of action if TR > 10	TR = IC_10,baseline_/IC_10_	Maeder et al. (2004)	Enhanced cytotoxicity of neuronal cells relative to baseline toxicity	This study
Specific effects relative to cytotoxicity	SR_cytotoxicity_ = IC_10_/EC_10_	Escher et al. (2020)	Neurite-specific: effects of neurite outgrowth inhibition relative to cytotoxicity	This study; Delp et al. (2021)
			DNT-specificity = EC_50_(viability)/EC_50_(neurite area)	Krug et al. (2013); Delp et al. (2018)
Specific effects relative to baseline toxicity	SR_baseline_ = IC_10,baseline_/EC_10_	Escher et al. (2020)	Neuronal-specific: effects of neurite outgrowth inhibition relative to baseline toxicity	This study; Delp et al. (2021)

A similar approach has been taken to calculate specific effects on target endpoints compared to either baseline toxicity or cytotoxicity for many different in vitro reporter gene assays (Escher et al. 2020). The specificity ratio, SR_cytotoxicity_, is the ratio between EC_10_ for a specific endpoint in a reporter gene assay and the experimental IC_10_ for cytotoxicity with Eq. 8

8 $${\text{SR}}_{\text{cytotoxicity}} = \frac{{{\text{IC}}_{{{10}}} }}{{{\text{EC}}_{{{10}}} }}.$$

In case of neurotoxicity addressed in the present study, we applied this equation using the EC_10_ of inhibition of neurite outgrowth and the IC_10_ for cytotoxicity toward differentiated neuronal cell lines. An analogous equation (Table 1) has been applied previously for the neurite outgrowth inhibition assay to identify “DNT-specific” effects (Delp et al. 2018; Masjosthusmann et al. 2020) or for identification of “neurite-specific” effects (Delp et al. 2021). Krug et al. (2013) defined a threshold of 4 to discriminate chemicals specifically acting on neurite outgrowth. We applied the same threshold of 4 for identification of “neurite-specific” effects using SR_cytotoxicity_

The specificity ratio, SR_baseline_, is the ratio of the effect concentration (EC_10_) and the associated predicted IC_10,baseline_ by Eq. 9

9 $${\text{SR}}_{\text{baseline}}\,\text{=}\,\frac{{\text{IC}}_{\text{10,baseline}}}{{\text{E}}{\text{C}}_{10}}.$$

SR_baseline_ can quantify how specifically chemicals can act on certain endpoints compared to minimal toxicity caused by baseline toxicity and this helps identify if specific MOAs contribute to the effects on the certain endpoints. According to Escher et al. (2020), SR_baseline_ ≤ 1 was considered as nonspecific, 1 ≤ SR_baseline_ < 10 as moderately specific (with high uncertainty), 10 ≤ SR_baseline_ < 100 as specific, and 100 ≤ SR_baseline_ as highly specific. For the purpose of the present study, we only used the threshold of SR_baseline_ of 10 to differentiate between nonspecific and specific effects. SR_baseline_ has not previously been applied for DNT. Delp et al. (2021) had used cytotoxicity in the U2OS osteosarcoma cell line as an indicator of nonspecific toxicity to identify “neuronal-specific” effects. We suggest that the predicted baseline toxicity in the same cell line measured under identical conditions (Lee et al. 2021) is an even better descriptor of nonspecific effects. The specific effects compared to baseline toxicity derived from SR_baseline_ will be referred as “neuronal-specific” toxicity henceforth to distinguish it from “neurite-specific” effects compared to cytotoxicity (SR_cytotoxicity_)

**Fig. 1:**
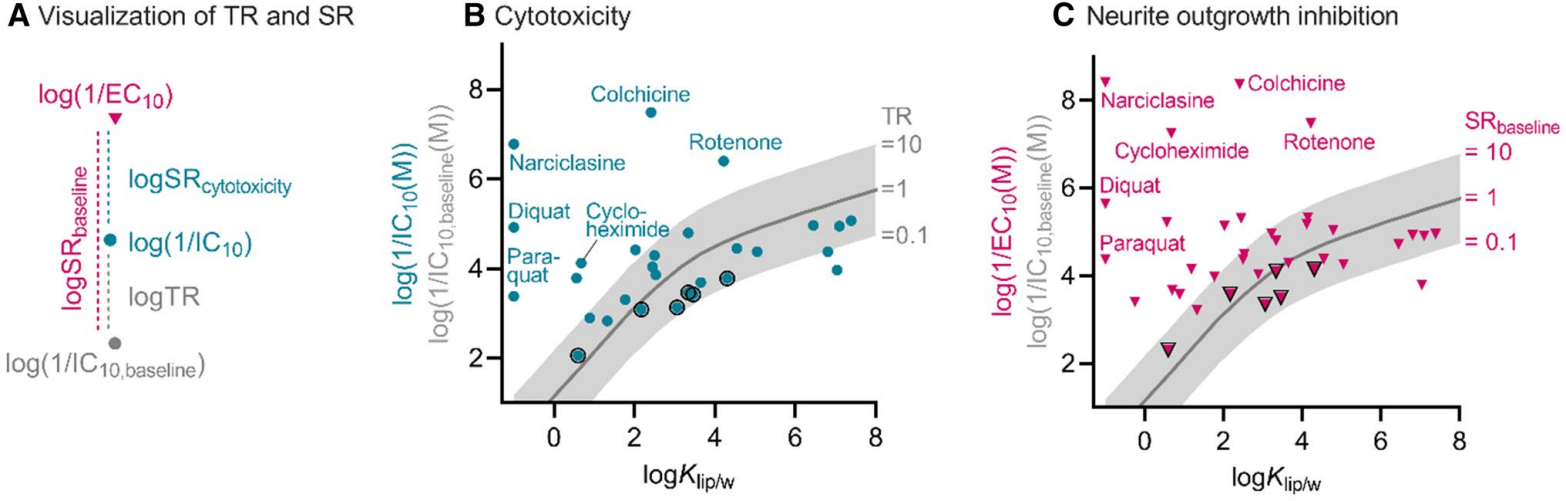
Inhibitory and effect concentrations against hydrophobicity of test chemicals. **A** Visualization of the toxic ratio TR (Eq. 7), the specificity ratios SR_cytotoxicity_ for neurite-specific effects (Eq. 8), and SR_baseline_ for neuronal-specific effects (Eq. 9). **B** Cytotoxicity as a function of the hydrophobicity expressed as liposome–water partition constants (*K*_lip/w_). The turquoise circles are the experimental inhibitory concentration for cytotoxicity (IC_10_; Table 2) with known baseline toxicants encircled in black. **(C)** Neurite outgrowth inhibition as a function of *K*_lip/w_. Magenta triangles indicate concentration leading to 10% reduction in neurite length (EC_10_; Table 2) which were experimentally determined in differentiated SH-SY5Y cells with known baseline toxicants encircled in black. Thick gray lines in both plots **B** and **C** correspond to predicted baseline toxicity causing 10% cytotoxicity (IC_10,baseline_; Eq. S1) as a function of *K*_lip/w_. The gray areas indicate when TR or SR_baseline_ is between 0.1 and 10

The terms “neurite-specific” SR_cytotoxicity_ and “neuronal-specific” SR_baseline_ allow one to differentiate between an enhanced effect caused by direct interference with neurite growth and those enhanced effects that are specific (such as mitochondrial toxicity) but not specific to neurites but affects the entire neuronal cell. Even chemicals that do not show neurite-specific effects can still show enhanced neurite degeneration compared to baseline toxicity due to neuronal-specific effects if SR_cytotoxicity_ < 4 and SR_baseline_ > 10. The highest tested concentration was used to calculate the upper limit of TR, TR_max_, and lower limits of SR_cytotoxicity,min_ for chemicals that only showed effects on neurite outgrowth and no cytotoxicity. The connection between effect concentrations and ratios is visualized in Fig. 1A, demonstrating that log SR_baseline_ = log SR_cytotoxicity_ + logTR.

## Results and discussion

### Assay performance

Endpoint-specific controls, that is, positive control chemicals for neurite outgrowth, showed high activity in the micromolar-to-nanomolar concentration range and were neurite-specific inhibitors of neurite outgrowth (Table 2). Narciclasine, the assay’s positive control, inhibited neurite outgrowth at the lowest EC_10_ showing the strongest effect potency among all tested chemicals. The selection of the endpoint-specific controls was originally based on specific effects on neurite outgrowth observed in LUHMES cells considering the ratio between EC_50_ and IC_50_ (Krug et al. 2013). The effect for all endpoint-specific controls (narciclasine, cycloheximide, colchicine, and rotenone) detected with the present experimental setup in SH-SY5Y cells corresponded well with cytotoxicity and neurite outgrowth inhibition observed in LUHMES cells by Krug et al. (2013), which confirmed the performance of our assay. Although EC_50_ values were derived for LUHMES cells and, therefore, slightly higher effect concentrations were reported than the corresponding IC_10_ or EC_10_ in SH-SY5Y cells, the effect concentrations for neurite outgrowth endpoint align within a factor of 10 (Fig. S3).

**Table 2:** Toxicity values for quantification of enhanced cytotoxicity (TR), neuronal-specific effects (SR_baseline_), and neurite-specific effects (SR_cytotoxicity_)

Group	Chemical name	Baseline toxicity			Cytotoxicity			Neurite outgrowth inhibition			TR	SR_baseline_	SR_cytotoxicity_	Group classification^e^	Remarks^f^
		log*K*_ow_^a^	log*K*_lip/w_^a^	IC_10,baseline_ (M)	IC_10_ (M)^b^	SE or CI^c^	Model^d^	EC_10_ (M)	SE or CI^c^	Model^d^					
Endpoint-specific controls	Narciclasine	− 1.2	− 1	1	1.7^.^10^–7^	1.6^.^10^–8^	L	3.9^.^10^–9^	3.6^.^10^–10^	L	6.0^.^10^6^	2.5^.^10^8^	42	1	
	Cycloheximide	0.6	0.7	1.0^.^10^–2^	7.7^.^10^–5^	[6.2^.^10^–5^, 9.5^.^10^–5^]	LL	5.6^.^10^–8^	7.7^.^10^–9^	L	136	1.9^.^10^5^	1370	1	Pl
	Colchicine	1.3	2.4	3.7^.^10^–4^	3.3^.^10^–8^	[2.2^.^10^–8^, 4.7^.^10^–8^]	LL	4.2^.^10^–9^	5.0^.^10^–10^	L	1.1^.^10^4^	8.7^.^10^4^	7.7	1	Pl
	Rotenone	4.1	4.2	3.2^.^10^–5^	4.0^.^10^–7^	4.1^.^10^–8^	L	3.4^.^10^–8^	4.3^.^10^–9^	L	81	948	11.7	1	
Baseline toxicants	2-Butoxyethanol	0.8	0.6	1.3^.^10^–2^	8.7^.^10^–3^	5.9^.^10^–4^	L	4.9^.^10^–3^	1.1^.^10^–3^	L	1.5	2.6	1.8	3	
	3-Nitroaniline	1.4	2.2	5.5^.^10^–4^	8.1^.^10^–4^	4.5^.^10^–5^	L	2.6^.^10^–4^	3.5^.^10^–5^	L	0.7	2.1	3.1	3	
	2-Allylphenol	2.6	3.1	1.4^.^10^–4^	7.3^.^10^–4^	3.6^.^10^–5^	L	4.5^.^10^–4^	4.5^.^10^–5^	L	0.2	0.3	1.6	3	
	4-Chloro-3-methylphenol	3.1	3.3	9.4^.^10^–5^	3.4^.^10^–4^	1.0^.^10^–5^	L	8.0^.^10^–5^	1.0^.^10^–5^	L	0.3	1.2	4.3	2	
	2-Phenylphenol	3.1	3.5	8.0^.^10^–5^	3.8^.^10^–4^	[3.4^.^10^–4^, 4.1^.^10^–4^]	LL	3.2^.^10^–4^	[2.8^.^10^–4^, 3.5^.^10^–4^]	LL	0.2	0.3	1.2	3	
	4-Pentylphenol	4.2	4.3	2.9^.^10^–5^	1.7^.^10^–4^	[1.6^.^10^–6^, 1.8^.^10^–4^]	LL	7.0^.^10^–5^	6.9^.^10^–6^	L	0.2	0.4	2.4	3	
AChE inhibitors	3-Hydroxycarbofuran	0.8	0.6	1.4^.^10^–2^	1.6^.^10^–4^	1.2^.^10^–5^	L	6.0^.^10^–6^	8.8^.^10^–7^	L	84	2283	27	1	
	Carbaryl	2.4	2.5	3.5^.^10^–4^	9.1^.^10^–5^	6.5^.^10^–6^	L	4.9^.^10^–6^	6.6^.^10^–7^	L	3.8	71	19	1	
	Diazoxon	2.1	0.9	6.5^.^10^–3^	1.3^.^10^–3^	1.5^.^10^–4^	L	2.7^.^10^–4^	2.6^.^10^–5^	L	5.1	24	4.7	1	
	Paraoxon-ethyl	2	1.8	1.1^.^10^–3^	4.9^.^10^–4^	3.9^.^10^–5^	L	1.1^.^10^–4^	1.1^.^10^–5^	L	2.2	11	4.7	1	
	Chlorpyrifos-oxon	3.3	2.5	3.0^.^10^–4^	1.4^.^10^–4^	[1.3^.^10^–4^, 1.4^.^10^–4^]	LL	3.3^.^10^–5^	3.8^.^10^–6^	L	2.2	9.1	4.2	2	
nAChR agonists	Thiamethoxam	− 0.1	− 0.3	1.1^.^10^–1^	I (3.1^.^10^–3^)		–	3.9^.^10^–4^	3.8^.^10^–5^	L	< 35	271	> 7.7	–	
	Imidacloprid	0.6	0.7	1.0^.^10^–2^	I (4.8^.^10^–4^)		–	2.1^.^10^–4^	2.7^.^10^–5^	L	< 21	47	> 2.3	–	
	Thiacloprid	1.3	1.2	3.4^.^10^–3^	I (1.2^.^10^–4^)		–	7.0^.^10^–5^	1.2^.^10^–5^	L	< 28	49	> 1.7	–	
	Acetamiprid	1.2	1.3	2.6^.^10^–3^	1.5^.^10^–3^	8.1^.^10^–5^	L	6.2^.^10^–4^	6.2^.^10^–5^	L	1.8	4.2	2.4	3	
	Clothianidin	0.7	2.9	1.8^.^10^–4^	I (2.8^.^10^–4^)		–	9.3^.^10^–5^	1.1^.^10^–5^	L	< 0.6	1.9	> 3.1	–	
GABA blockers	Fipronil	4.0	2.5	3.2^.^10^–4^	5.2^.^10^–5^	[5.1^.^10^–5^, 5.3^.^10^–5^]	LL	4.3^.^10^–5^	–	B	6.2	7.5	1.2	3	Pr
	Fipronil sulfone	3.2	3.3	9.4^.^10^–5^	1.6^.^10^–5^	[1.5^.^10^–5^, 1.7^.^10^–5^]	LL	1.6^.^10^–5^	-	B	5.9	5.9	1.0	3	Pr
	α-Endosulfan	3.8	4.6	2.2^.^10^–5^	3.5^.^10^–5^	[3.2^.^10^–5^, 3.8^.^10^–5^]	LL	4.2^.^10^–5^	-	B	0.6	0.5	0.8	3	Pr
	Dieldrin	5.4	5.1	1.4^.^10^–5^	4.1^.^10^–5^	3.0^.^10^–6^	L	5.5^.^10^–5^	-	B	0.3	0.2	0.7	3	Pr
Sodium channel agonists	Bifenthrin	6.8	6.5	4.3^.^10^–6^	1.1^.^10^–5^	6.5^.^10^–7^	L	2.0^.^10^–5^	–	B	0.4	0.2	0.6	3	
	4,4'-DDT	6.9	7.1	2.8^.^10^–6^	1.1^.^10^–5^	9.1^.^10^–7^	L	1.2^.^10^–5^	–	B	0.2	0.2	0.9	3	Pr
Mitochondrial toxicants	Azoxystrobin	2.5	2.0	7.0^.^10^–4^	3.8^.^10^–5^	2.1^.^10^–6^	L	7.4^.^10^–6^	1.9^.^10^–6^	L	18	94	5.1	1	
	Picoxystrobin	3.6	3.2	1.1^.^10^–4^	I (7.3^.^10^–5^)		–	1.1^.^10^–5^	2.1^.^10^–6^	L	< 1.5	10	> 6.7	–	
	Fluoxastrobin	4.0	4.1	3.6^.^10^–5^	I (3.6^.^10^–5^)		–	6.6^.^10^–6^	9.4^.^10^–7^	L	< 1.0	5.4	> 5.4	–	
	Pyraclostrobin	4.0	4.1	3.5^.^10^–5^	I (3.5^.^10^–5^)		–	4.7^.^10^–6^	8.8^.^10^–7^	L	< 1.0	7.4	> 7.4	–	
	Trifloxystrobin	4.5	4.8	1.8^.^10^–5^	I (3.2^.^10^–5^)		–	9.2^.^10^–6^	2.5^.^10^–6^	L	< 0.6	1.9	> 3.4	–	
	Hexachlorophene	7.5	6.8	3.4^.^10^–6^	4.2^.^10^–5^	3.5^.^10^–6^	L	1.2^.^10^–5^	–	B	0.1	0.3	3.5	3	
Redox cyclers	Paraquat	–1.8	− 1	1	4.2^.^10^–4^	[2.8^.^10^–4^, 6.0^.^10^–4^]	LL	4.2^.^10^–5^	6.1^.^10^–6^	L	2400	2.4^.^10^4^	9.8	1	Pl
	Diquat	− 2.0	− 1	1	1.2^.^10^–5^	[9.0^.^10^–6^, 1.6^.^10^–5^]	LL	2.3^.^10^–6^	2.6^.^10^–7^	L	8.3^.^10^4^	4.4^.^10^5^	5.3	1	Pl
Endocrine disruptors	Bisphenol A	3.3	3.7	6.3^.^10^–5^	2.0^.^10^–4^	2.7^.^10^–5^	L	5.1^.^10^–5^	6.7^.^10^–6^	L	0.3	1.2	4.0	3	
	3,3′,5,5′-Tetra- bromobisphenol A	6.7	7.0	2.9^.^10^–6^	1.1^.^10^–4^	7.0^.^10^–6^	L	1.6^.^10^–4^	–	B	0.03	0.02	0.7	3	
	Di(2-ethylhexyl) phthalate	7.5	7.4	2.3^.^10^–6^	8.5^.^10^–6^	[6.7^.^10^–6^, 1.0^.^10^–5^]	LL	1.1^.^10^–5^	[8.8^.^10^–6^, 1.4^.^10^–5^]	LL	0.3	0.2	0.8	3	Pr

^a^log*K*_ow_ and log*K*_lip/w_ were derived as described in Lee et al. (2021)

^b^I: Inactive; highest tested concentration (M) was given in brackets

^c^Standard error (SE) for IC_10_ or EC_10_ in linear regression; 95% confidence interval (CI) for IC_10_ or EC_10_ in log-logistic model

^d^L: linear regression; LL: log-logistic model; B: Brain–Cousens model

^e^Group 1: SR_cytotoxicity_ > 4, SR_baseline_ > 10; group 2: SR_cytotoxicity_ > 4, SR_baseline_ < 10; group 3: SR_cytotoxicity_ < 4, SR_baseline_ < 10

^f^Pr: precipitation/turbidity observed; Pl: Plateau observed in concentration–response curves for cell viability

It is remarkable that neurite-specific inhibitors were also highly neuronal-specific, that is, their TR and SR_baseline_ were also very high. Only for cycloheximide neurite-specific effects dominated over neuronal-specific effects. Narciclasine, in contrast, had a TR of 6 million, which means that it is highly toxic to neuronal cells, but the specific effect on neurite outgrowth is moderate compared to this with a SR_cytotoxicity_ of 42.

### Effects in relation to hydrophobicity of the chemicals

IC_10_ for cytotoxicity and EC_10_ for neurite outgrowth inhibition or stimulation were determined with best-fit model parameters (Table S3) from the CRCs (Fig. S4). The effect concentrations are given with the applied CRC model, calculated ratios, classification, and experimental issues due to turbidity/precipitation for all individual chemicals in Table 2. The IC_10_ (Fig. 1B) and EC_10_ (Fig. 1C) were plotted against the hydrophobicity expressed as log*K*_lip/w_ and compared with predictions for IC_10,baseline_ calculated with the baseline cytotoxicity QSAR (Eq. S1; Table 2).

Many chemicals had TRs, a measure for enhanced cytotoxicity, between 0.1 and 10 and were classified as baseline toxicants in SH-SY5Y cells (Fig. 1B). Among 37 chemicals, 70% were baseline toxicants (26 chemicals including the 6 known baseline toxicants). The remaining 11 chemicals included the 4 endpoint-specific controls and their TR exceeding 10 indicated that specific MOAs rather than baseline toxicity could be involved in cytotoxicity. The analysis for diquat and paraquat is highly uncertain, because they have double cationic charges, are very hydrophilic, and are therefore outside the applicability domain of the baseline cytotoxicity QSAR (Lee et al. 2021). Their log*K*_lip/w_ was assumed to be -1 as for other very hydrophilic chemicals (Gobas et al. 1988). This estimate still gave highly elevated cytotoxicity with TR > 10^3^, which is reasonable given that they act as redox cyclers forming radicals and reactive oxygen species (Bonneh-Barkay et al. 2005; Conning et al. 1969). When we had a closer look at pesticides (24 pesticides except endpoint-specific controls), 71% of them had TRs of baseline toxicants. The chemicals with TR > 10 were mostly observed for chemicals with log*K*_lip/w_ < 4, and therefore, TR was more likely to be higher for hydrophilic chemicals.

Similar trends with respect to hydrophobicity were observed for neuronal-specific effects, i.e., the ratio of IC_10,baseline_ to the EC_10_ for neurite outgrowth inhibition (Fig. 1C). Neuronal-specific effects were again mostly observed for hydrophilic chemicals with log*K*_lip/w_ < 4. SR_baseline_ ranged from 0.02 to 2.5  ×10^8^, and 41% of the tested chemicals exceeded SR_baseline_ of 10, which is only 11% more than those that exceeded TR of 10. When it comes to neurite-specific effects, SR_cytotoxicity_ ranged from 0.6 to 1370, and high specificity was observed especially for endpoint-specific controls, carbamates, and redox cyclers (Table 2).

Hydrophobic chemicals were mostly classified as baseline cytotoxicants and, hence, appear more likely to trigger both cytotoxicity and neurite outgrowth inhibition through baseline toxicity (Fig. 1). However, they are still very potent due to their high hydrophobicity and were with the lowest EC_10_ and IC_10_ among the pesticides. Apart from the known baseline toxicants, 63% of chemicals that did not act neuronal-specific (SR_baseline_ < 10) exceeded a log*K*_lip/w_ of 4, and IC_10_ and EC_10_ for these chemicals were close to IC_10,baseline_.

### Enhanced effects over baseline cytotoxicity (TR and SR_baseline_)

**Fig. 2:**
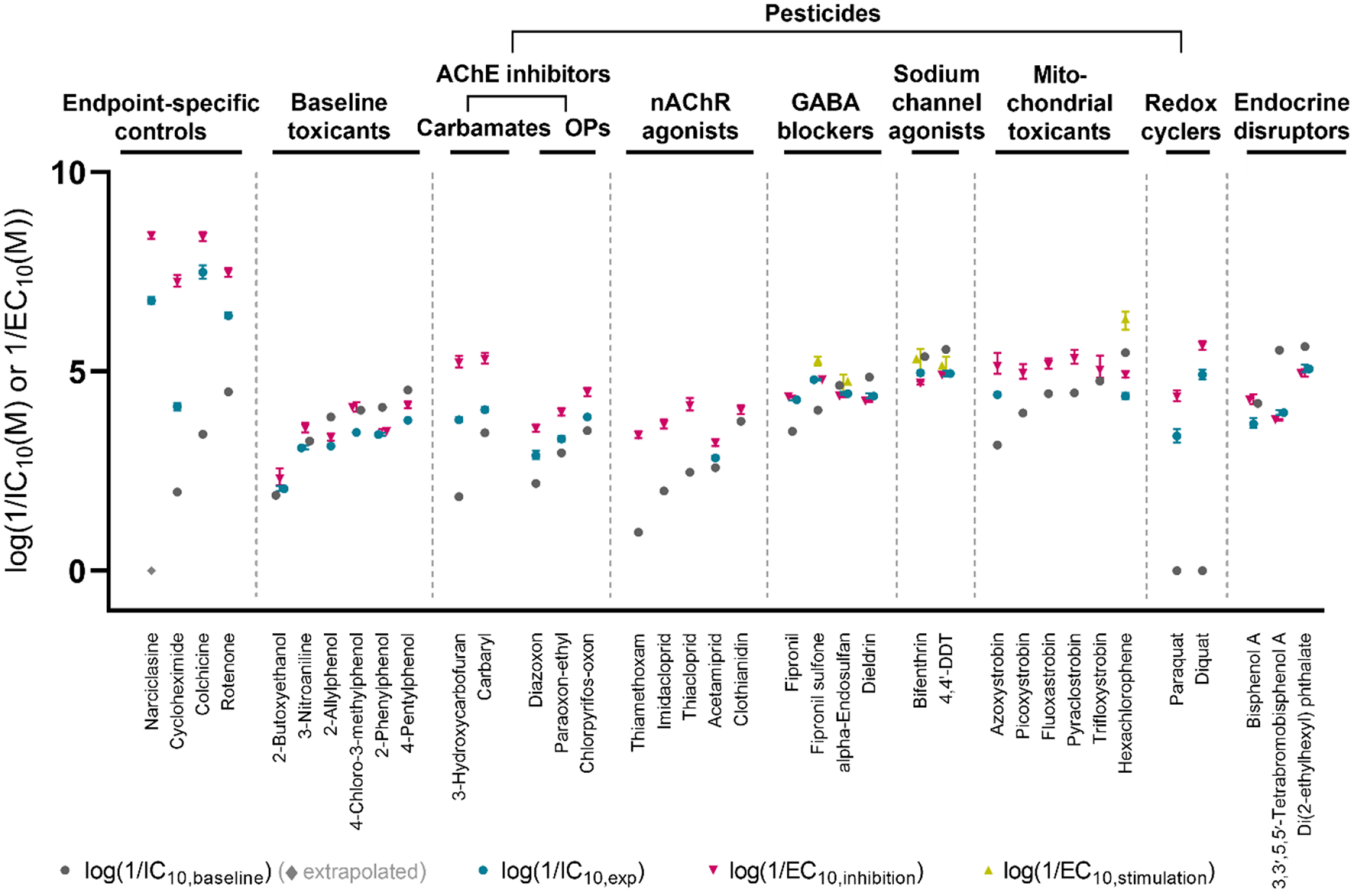
Effect concentrations for baseline toxicity, cytotoxicity, and neurite outgrowth inhibition or stimulation sorted by MOA class. IC_10,baseline_ for baseline toxicity (extrapolated for very hydrophilic chemicals), IC_10,exp_ for cytotoxicity, and EC_10_ for inhibiting or stimulating effects on neurite outgrowth in different groups of chemicals were shown in the order of increasing *K*_lip/w_ within each MOA class. The test chemicals include endpoint-specific controls for neurite outgrowth inhibition (Aschner et al. 2017), known baseline toxicants (Vaes et al. 1998), pesticides with diverse mode of action grouped into the MOA classes of acetylcholinesterase (AChE) inhibitors, nicotinic acetylcholine receptor (nAChR) agonists, γ-aminobutyric acid (GABA)-gated chloride channel blockers, voltage-gated sodium channel agonists, mitochondrial toxicants, redox cyclers, and endocrine disruptors. The error bars represent the 95% confidence intervals; in case of very small confidence intervals, error bars are hidden by the symbol

The tested chemicals were categorized into nine MOA classes, and their IC_10,baseline_, IC_10_, and EC_10_ were grouped in Fig. 2 by their MOA classes with an increasing *K*_lip/w_ within each class.

The four endpoint-specific controls were extremely neuronal-specific and showed highly enhanced cytotoxicity (Fig. 2). Their effect potency considering nominal concentration was the highest among the MOA groups. TR ranged from 81 to 6.0 × 10^6^ and SR_baseline_ ranged from 948 to 2.5 × 10^8^ for this group of chemicals. Narciclasine, a toxic alkaloid found in *Amaryllidaceae* plants, showed the most neuronal-specific effects (SR_baseline_ = 2.5 × 10^8^) and its cytotoxicity also enhanced the most over baseline toxicity (TR = 6.0 × 10^6^) among all tested chemicals. Cycloheximide was also extremely neuronal-specific, which are more contributed by specific effects on neurite outgrowth than by cytotoxicity considering SR_cytotoxicity_ > TR. In contrast, the neuronal-specific effects were more contributed by enhanced cytotoxicity than specific effects on neurite outgrowth for plant-derived alkaloid colchicine and isoflavone rotenone. The endpoint-specific controls are all naturally occurring toxic substances but also have been synthesized, and cycloheximide and rotenone were used as pesticides (Richardson et al. 2019).

All well-known baseline toxicants (2-butoxyethanol, 3-nitroaniline, 2-allylphenol, 4-chloro-3-methylphenol, 2-phenylphenol, and 4-pentylphenol), which are industrial chemicals, were confirmed as baseline toxicants with respect to cytotoxicity as well as neurite outgrowth inhibition (Fig. 2). They all showed no enhanced cytotoxicity compared to baseline toxicity with TR from 0.2 to 1.5 and no neuronal-specific effects SR_baseline_ from 0.3 to 2.6, which proved our assay quality as negative controls. Despite all of them lacking specific MOAs, they differed in the effect potency by a factor of 50 for cytotoxicity (IC_10_) and 70 for neurite outgrowth inhibition (EC_10_) due to the variation in hydrophobicity.

Neuronal-specific toxicity was mostly accompanied by enhanced cytotoxicity, which led to highly elevated effect potency for inhibition of neurite outgrowth compared to baseline toxicity (Fig. 2). Among the pesticides, carbamates (3-hydroxycarbofuran and carbaryl) and redox cyclers (paraquat and diquat) showed high neuronal specificity, and high TRs were also observed for these two groups except carbaryl. This means that these pesticides are neurotoxic, but that inhibition of neurite outgrowth is not the cause but a consequence of their neurotoxic effect triggered by another initiating event, such as potentially mitochondrial toxicity.

The hydrophobicity-dependent trends were maintained within the same MOA group for the pesticides as TR and SR_baseline_ tended to increase with decreasing hydrophobicity within the group. However, except carbamates and redox cyclers, the pesticides with determined IC_10_ were mostly classified as baseline toxicants for the SH-SY5Y cells. The two hydrophobic chemical groups (all with log*K*_lip/w_ > 4), GABA receptor blockers (fipronil, fipronil sulfone, α-endosulfan, and dieldrin), and sodium channel agonists (bifenthrin, 4,4′-DDT) did not exceed TR nor SR thresholds, and thus classified as baseline toxicants for the tested endpoints. Highly hydrophobic chemicals such as pyrethroids other than bifenthrin, PAHs, PCBs, and PBDEs were inactive up to IC_10, baseline_ (Table S2). It has often been observed that very hydrophobic chemicals are highly toxic, but do not show any excess toxicity over baseline (Escher and Hermens 2002). This implies that they do not bind to specific receptors and/or that accumulation in membranes is the dominant process.

EDCs were tested to evaluate DNT effects of typical environmental chemicals that do not have any primary neurotoxic MOAs. All three EDCs from the NTP library were classified as baseline toxicants and did not show enhanced cytotoxicity or neuronal-specific toxicity in SH-SY5Y cells (Fig. 2). The experimental effect concentrations for 3,3′,5,5′-tetrabromobisphenol A were higher than the expected baseline toxicity possibly due to uncertainty in predicted IC_10,baseline_ for anionic chemicals (Lee et al. 2021).

Neuronal-specific effects can be caused not only by specific MOA affecting neurite outgrowth directly but also by enhanced cytotoxicity. The latter case is not neurite-specific, since their effects on neurite outgrowth just resulted from adverse effects on overall cell health, which necessitates quantification of neurite-specific effects in the following section.

### Neurite-specific effects (SR_cytotoxicity_)

For neurite-specific effects, a threshold of 4 was used to define the specific effects on neurite outgrowth compared to cytotoxicity (SR_cytotoxicity_ > 4), which was proposed by Krug et al. (2013) and confirmed independently by our calculation (Text S2; Table S4). All chemicals with SR_cytotoxicity_ > 4 had EC_10_ which clearly distinguished from IC_10_ considering the overlap of their average + 3 standard deviation or their confidence interval given in Table 2.

All four endpoint-specific controls had neurite-specific effects (Fig. 2). SR_cytotoxicity_ ranged from 7.7 to 1370 for these chemicals, which are all above the defined threshold. Our assay control, narciclasine, inhibited neurite outgrowth specifically (SR_cytotoxicity_ = 42) possibly by activation of Rho signaling pathway which regulates actomyosin contractility. Cycloheximide inhibits protein synthesis by interfering with translocation step, and showed the most specific effects on neurite outgrowth inhibition among all the tested chemicals with SR_cytotoxicity_ of 1370. Colchicine, a microtubule polymerization inhibitor, and rotenone, a mitochondrial toxicant, showed relatively moderate specificity with SR_cytotoxicity_ of 7.7 and 11.7, respectively.

As expected, the six known baseline toxicants all showed nonspecific effects on neurite outgrowth with SR_cytotoxicity_ from 1.2 to 3.1, except for 4-chloro-3-methylphenol having SR_cytotoxicity_ slightly over the threshold (4.3).

AChE inhibitors, which are used as insecticides, showed different patterns depending on their interaction at the target site (Fig. 2). Carbamates bind reversibly to AChE to disturb the enzymatic function, while organophosphates (OP) bind irreversibly (Colovic et al. 2013), and both undifferentiated and differentiated SH-SY5Y cells are known to express AChE (de Medeiros et al. 2019). The two reversible AChE inhibitors, 3-hydroxycarbofuran and carbaryl, showed SR_cytotoxicity_ > 10 and their specificity and effect potency for neurite outgrowth inhibition were the highest among the tested pesticides. For the three irreversible AChE inhibitors, SR_cytotoxicity_ stayed fairly constant at around 4.5, close to the SR_cytotoxicity_ threshold of 4. The role of AChE in neurite outgrowth has been reviewed, and can be explained by both enzymatic and non-enzymatic way (Paraoanu and Layer 2008). It was described that secreted acetylcholine could signal to AChE of adjacent cells to direct neurite outgrowth, while AChE also could directly support neurite outgrowth by structural interaction with extracellular matrix protein such as laminin. However, it should be still elucidated whether reversible and irreversible AChE inhibitors could behave differently in these processes.

Other specific MOAs may exist for carbamates which caused specific effects on neurite outgrowth with minor effects on cell viability. While mechanistic understanding remains limited for DNT, it has been reported that impairment of signaling pathways can disturb neurodevelopmental processes including neurite outgrowth (Bal-Price et al. 2018; Masjosthusmann et al. 2020). The interaction with signaling pathways may also be responsible for effects on differentiation of cells and it has been reported that carbofuran impaired neuronal differentiation through transforming growth factor beta (TGF-β) signaling, which mediates neurogenesis, in rat hippocampus (Seth et al. 2017). This observation can explain our results as we tested cells in early differentiation stage with short-term differentiation compared to the previous studies (Constantinescu et al. 2007; Shipley et al. 2016).

The mitochondrial toxicants are all applied as fungicides in agriculture and showed broad specificity of their effects on neurite outgrowth, although these pesticides commonly target mitochondrial respiration representing a basal function of all cells (Fig. 2). Rotenone, one of the endpoint-specific controls, showed specific effects despite of its high hydrophobicity, which suggests that specific toxicity can still manifest if the MOA is highly specific. Other mitochondrial toxicants, strobilurins and hexachlorophene, showed relatively low specific effects. All strobilurins with exception of trifloxystrobin had moderate SR_cytotoxicity_ above 4, and hexachlorophene was nonspecific. This variety in neurite-specific effects could be explained by difference in their MIEs (Delp et al. 2019). Delp et al. (2019) investigated the specific effects of mitochondrial toxicants on neurite outgrowth inhibition and their link to MIEs in LUHMES cells. They observed that rotenone showed highly neurite-specific effects and targeted complex I in mitochondrial respiratory chain, and the other complex I inhibitors commonly showed relatively high neurite-specific effects. In contrast, they found that the strobilurins acted as complex III inhibitors and hexachlorophene was a phenolic uncoupler of oxidative phosphorylation. Both strobilurins and hexachlorophene showed less neurite-specific effects in the study of Delp et al. (2019), which agrees well with our observation.

The redox cyclers diquat and paraquat showed moderate neurite-specific effects, which were accompanied by highly enhanced cytotoxicity (Fig. 2). This indicates that their specific MOAs can contribute not only to neurite-specific effects but also strongly to neuronal-specific and cytotoxic effects. Diquat and paraquat are photosynthesis inhibitors, and were historically applied as herbicides, but have been phased out as plant protection products (Conning et al. 1969). Both were considered as endpoint-specific controls by Aschner et al. (2017), and we not only confirmed their neurite-specific effects in our assay but also brought more details in that their effect is highly enhanced over baseline toxicity. Redox cycling and the subsequent production of reactive oxygen species can generally impair cell health, but this can also possibly explain the specific effects on neurite outgrowth as it has been reported that cytoskeleton dynamics can be regulated by oxidative species in neuronal cells and the redox imbalance can affect neurite outgrowth (Wilson and Gonzalez-Billault 2015).

### Stimulating effects

Two endpoint-specific controls for stimulating neurite outgrowth (Aschner et al. 2017) confirmed the capacity of our assay to also capture stimulating effects (Text S3, Table S5, Fig. S5). Stimulating effects over 150% were observed for both HA-1077 and Y-27632 and their hormetic parameter *f* was significant (*p* < 0.05).

All GABA receptor blockers, all sodium channel agonists, hexachlorophene, and 3,3′,5,5′-tetrabromobisphenol A stimulated neurite outgrowth and gave significant parameter *f* with *p* < 0.05 (Fig. 2, Table S6). Especially, hexachlorophene showed the most distinct stimulating effects considering its highest hormesis effect parameter *f*. However, EC_10_ for stimulating effects could only be derived for five chemicals (fipronil sulfone, α-endosulfan, bifenthrin, 4,4′-DDT, and hexachlorophene) and the EC_10_ values for stimulating effects are given in Table S6. The best-fit curves in Brain–Cousens model did not reach 110% level in neurite length for the rest of chemicals (Fig. S4), and therefore, the stimulating effects could not be quantified. To confirm that the stimulating effects were not due to increased cell number, total neurite length divided by total cell count was compared, and the parameter *f* stayed significant and gave comparable values to the original analysis (Table S6).

The stimulating effects were observed mostly for the chemicals interacting with ion channels. The GABA receptor and sodium channels can be involved in stimulating neurite outgrowth as reported previously (Davis et al. 2004; Michler 1990), but the relevant literature to explain the stimulating effects is still limited and the effects have been rarely quantified. Furthermore, it should be noted that the observed stimulating effects could reflect general stress responses given that they occurred close to concentrations causing cytotoxicity and the hormesis parameter *f* was not high, except for hexachlorophene. Also, considering that the stimulating effects were followed by the inhibiting effects close to cytotoxic level, the stimulating effects can be masked by the cytotoxic effects and might be captured more sensitively from long-term exposure at low concentration. For example, clothianidin showed stimulating effects in differentiated SH-SY5Y cells after co-exposure to brain-derived neurotrophic factor for 3 days (Hirano et al. 2019), while we did not observe any stimulating effects for this chemical in our experimental set up.

### Classification based on SR_baseline_ and SR_cytotoxicity_

**Fig. 3:**
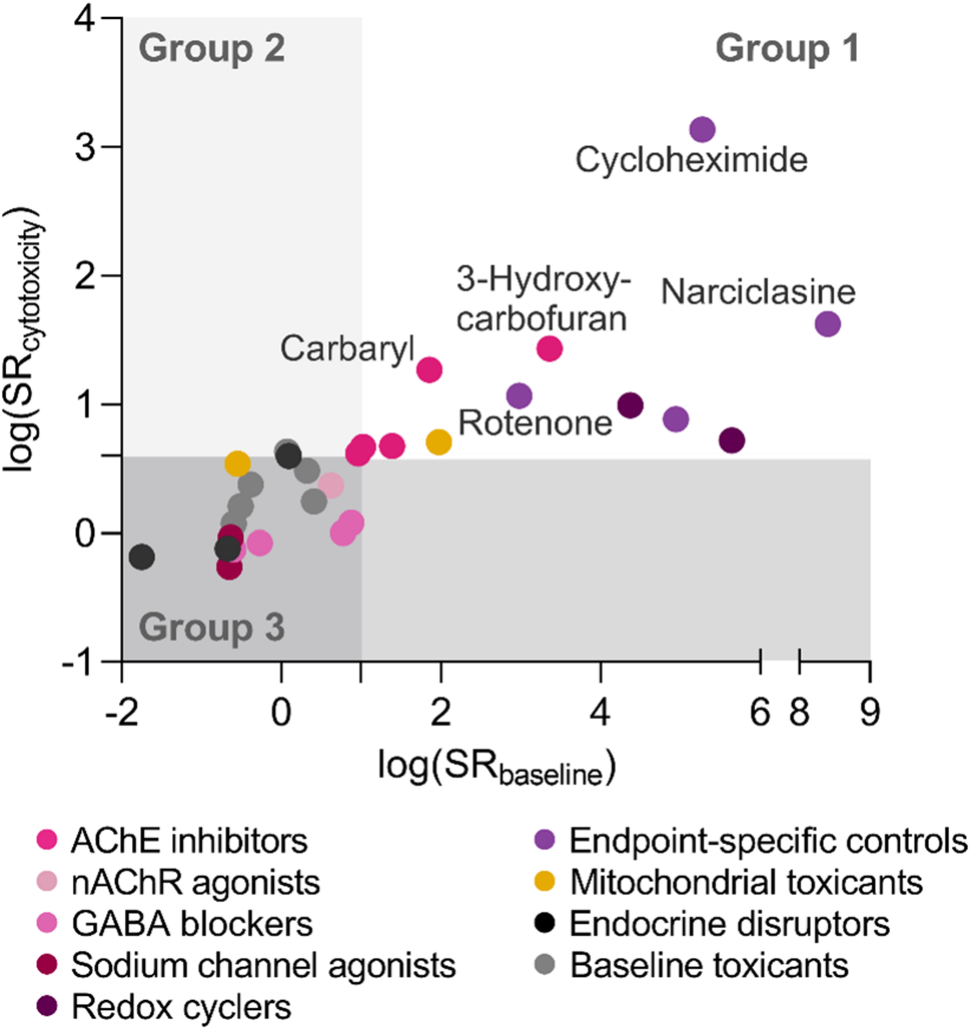
Classification of test chemicals based on their specificity ratios SR. Neuronal-specific effects were explained by SR_baseline_, and neurite-specific effects were explained by SR_cytotoxicity_. Based on SR_baseline_ and SR_cytotoxicity_, the test chemicals were classified into three groups: neurite-specific and neuronal-specific chemicals (group 1; SR_cytotoxicity_ > 4 and SR_baseline_ > 10), chemicals with only neurite-specific effects (group 2; SR_cytotoxicity_ > 4 and SR_baseline_ < 10), and baseline toxicants (SR_cytotoxicity_ < 4 and SR_baseline_ < 10)

The test chemicals were categorized into three groups based on neurite- and neuronal-specific toxicity regarding to neurite outgrowth inhibition in Fig. 3: neurite-specific and neuronal-specific chemicals in group 1 (SR_cytotoxicity_ > 4, SR_baseline_ > 10), exclusively neurite-specific chemicals without enhanced cytotoxicity in group 2 (SR_cytotoxicity_ > 4, SR_baseline_ < 10), and baseline toxicants in group 3 (SR_cytotoxicity_ < 4, SR_baseline_ < 10). Chemicals in group 1 are likely to affect cell viability and neurite outgrowth through specific MOAs other than baseline toxicity, while specific MOAs can mainly contribute to neurite outgrowth inhibition with lower effects on cell viability for group 2 chemicals. No chemicals were found in a fourth group that would be neuronal-specific but not neurite-specific.

The majority of chemicals fell into group 1 or 3 (Fig. 3) and the highly neurite-specific effects of group 1 chemicals are prone to accompany elevated cytotoxicity as described above. Both neurite- and neuronal-specific effects were mainly observed for endpoint-specific controls and AChE inhibitors. Endpoint-specific controls were confirmed to show specific effects on neurite outgrowth and our novel analysis also showed that they have even more pronounced enhanced cytotoxicity with SR_baseline_ > SR_cytotoxicity_ (Fig. 3). The same applied for AChE inhibitors with rather distinct SR values for the carbamates, while the OPs had much lower SR values close to the threshold.

Only a few chemicals were classified into group 2, and the group 2 chemicals can have high uncertainty in their classification as their SRs laid closely to the threshold. One of baseline toxicants, 4-chloro-3-methylphenol, was included in group 2, but its SR_cytotoxicity_ (4.3) is just above the threshold and can be classified differently considering its standard error. Therefore, the chemicals close to the threshold must be regarded with caution as there can be some uncertainty in the definition of the thresholds and their classification can be improved by refining the threshold based on a larger training set of chemicals without specific effects.

## Conclusions and outlook

The proposed approach considering both neurite-specific and neuronal-specific effects in the neurite outgrowth assay provides new information that complements the current DNT in vitro testing strategies. On one hand, the specificity ratio SR_cytotoxicity_ can identify chemicals with neurite-specific DNT effects and, therefore, can be used to prioritize test chemicals for further testing. Hereby, we identified two carbamates, 3-hydroxycarbofuran and carbaryl, as highly neurite-specific chemicals in SH-SY5Y cells. On the other hand, SR_baseline_ can be used to identify neurotoxic chemicals whose neurotoxicity is not driven by specific inhibition of neurite outgrowth. Furthermore, SR_baseline_ may serve as a useful measure when comparing effect potency of a given chemical between different cell models as the current DNT in vitro testing strategies utilize multiple cell models with diverse endpoints. It can also support estimation of specificity in case that no cytotoxicity was observed by replacing the use of the highest test concentration or a factor thereof as reference level (Delp et al. 2018). These two specificity ratios can clarify if the effects are triggered by their specific MOAs or merely by baseline toxicity arising from their high hydrophobicity and strong toxic effects can be observed at low concentration for hydrophobic chemicals due to their membrane affinity. Therefore, while cytotoxicity is considered as a reference to identify neurite-specific effects, baseline toxicity provides an anchor to compare the observed toxic effects for individual endpoints.

Mechanistic research underlying specific effects can help build a clear connection between MIEs and adverse outcomes in DNT and expand knowledge of MOAs (Carlson et al. 2020). Other key neurodevelopmental processes such as cell migration could potentially be more sensitive DNT endpoints than neurite outgrowth, and therefore, a battery of endpoints can capture DNT effects more comprehensively (Behl et al. 2019; Harrill et al. 2018; Masjosthusmann et al. 2020). As for our observation on neurite outgrowth, primary MOAs of the pesticides are not necessarily the only specific MOA involved in cytotoxicity and inhibition of neurite outgrowth. The insecticides are usually less potent in mammals due to species specificity and they have secondary targets which can possibly induce toxic effects in non-target organisms (Lushchak et al. 2018). Therefore, multiple MOAs, which can be primary MOA or other secondary MOAs, might contribute to the observed inhibition of neurite outgrowth and cytotoxicity. In case of hydrophobic chemicals, these specific MOAs even can compete with baseline toxicity and baseline toxicity can prevail over the specific MOA for more hydrophobic chemicals due to their high affinity to membranes (Escher and Hermens 2002).

In terms of in vitro models for DNT, although SH-SY5Y cells have been widely used as a model to study neurite outgrowth, their abnormal physiology (Do et al. 2007) originated from tumor origin could limit the interpretation of the observed toxic effects in this model. Therefore, comparison of the effects with those from different models can improve the reliability of this model. LUHMES cells can be applied for this purpose and also can serve as a proper tool to test the effects on neurite outgrowth considering their non-oncogenic human origin. Furthermore, more biologically relevant exposure scenario can be achieved by testing potential DNT chemicals in co-culture with astrocytes.

## Supplementary Information

Below is the link to the electronic supplementary material.

Supplementary file1 (DOCX 3673 KB)
