# Supplementary material for: Inhibition of neurite outgrowth and enhanced effects compared to baseline toxicity in SH-SY5Y cells
Source: Arch Toxicol. 2022 Feb 19;96(4):1039–53. doi: 10.1007/s00204-022-03237-x (PMC8921145; doi:10.1007/s00204-022-03237-x)
Supplement: Supplementary file 1 — Supplementary file1 (DOCX 3673 KB) [file 204_2022_3237_MOESM1_ESM.docx]

**Supplementary information**

**Inhibition of neurite outgrowth and enhanced effects compared to baseline toxicity in SH-SY5Y cells**

Jungeun Lee^1^, Beate I. Escher^1,2^, Stefan Scholz^3^ and Rita Schlichting^1^

^1^Department of Cell Toxicology, Helmholtz Centre for Environmental Research – UFZ, Leipzig, Germany

^2^Environmental Toxicology, Center for Applied Geoscience, Eberhard Karls University Tübingen, Tübingen, Germany

^3^Department of Bioanalytical Toxicology, Helmholtz Centre for Environmental Research – UFZ, Leipzig, Germany

## **Table S1. Additional information of the tested chemicals.**

| **Chemical class or use class** | **MOA class** | **Chemical name** | **CAS number** | **Supplier** |
| --- | --- | --- | --- | --- |
| Natural product | Endpoint-specific controls | Narciclasine | 29477-83-6 | Roth |
| Natural product | Endpoint-specific controls | Colchicine | 64-86-8 | Sigma-Aldrich |
| Natural product | Endpoint-specific controls | Cycloheximide | 66-81-9 | Sigma-Aldrich |
| Natural product | Endpoint-specific controls | Rotenone | 83-79-4 | Sigma-Aldrich |
| - | Endpoint-specific controls | HA-1077 dihydrochloride | 203911-27-7 | Sigma-Aldrich |
| - | Endpoint-specific controls | Y-27632 dihydrochloride | 129830-38-2 | Sigma-Aldrich |
| Insecticide | AChE inhibitors | Chlorpyrifos-oxon | 5598-15-2 | Dr. Ehrenstorfer |
| Insecticide | AChE inhibitors | Diazoxon | 962-58-3 | HPC Standards |
| Insecticide | AChE inhibitors | Paraoxon-ethyl | 311-45-4 | Sigma-Aldrich |
| Insecticide | AChE inhibitors | Carbaryl | 63-25-2 | Sigma-Aldrich |
| Insecticide | AChE inhibitors | 3-Hydroxycarbofuran | 16655-82-6 | Sigma-Aldrich |
| Insecticide | nAChR agonists | Acetamiprid | 135410-20-7 | HPC Standards |
| Insecticide | nAChR agonists | Clothianidin | 210880-92-5 | Sigma Aldrich |
| Insecticide | nAChR agonists | Imidacloprid | 138261-41-3 | Sigma-Aldrich |
| Insecticide | nAChR agonists | Thiacloprid | 111988-49-9 | Fluka |
| Insecticide | nAChR agonists | Thiamethoxam | 153719-23-4 | HPC Standards |
| Insecticide | Sodium channel agonists | Acrinathrin | 101007-06-1 | Dr. Ehrenstorfer |
| Insecticide | Sodium channel agonists | Bifenthrin | 82657-04-3 | Sigma Aldrich |
| Insecticide | Sodium channel agonists | α-Cypermethrin | 67375-30-8 | Sigma-Aldrich |
| Insecticide | Sodium channel agonists | Cyfluthrin | 68359-37-5 | Dr. Ehrenstorfer |
| Insecticide | Sodium channel agonists | λ-Cyhalothrin | 91465-08-6 | Sigma-Aldrich |
| Insecticide | Sodium channel agonists | Deltamethrin | 52918-63-5 | Sigma-Aldrich |
| Insecticide | Sodium channel agonists | Esfenvalerate | 66230-04-4 | Sigma-Aldrich |
| Insecticide | Sodium channel agonists | Permethrin | 52645-53-1 | HPC Standards |
| Insecticide | GABA receptor blockers | 4,4'-DDT | 50-29-3 | Sigma-Aldrich |
| Insecticide | GABA receptor blockers | Dieldrin | 60-57-1 | Sigma-Aldrich |
| Insecticide | GABA receptor blockers | α-Endosulfan | 959-98-8 | Fluka |
| Insecticide | GABA receptor blockers | β-Endosulfan | 33213-65-9 | HPC Standards |
| Insecticide | GABA receptor blockers | Endosulfan sulfate | 1031-07-8 | Sigma-Aldrich |
| Insecticide | GABA receptor blockers | Fipronil | 120068-37-3 | Sigma-Aldrich |
| Insecticide | GABA receptor blockers | Fipronil sulfone | 120068-36-2 | HPC Standards |
| Fungicide | Mitochondrial toxicants | Azoxystrobin | 131860-33-8 | Sigma-Aldrich |
| Fungicide | Mitochondrial toxicants | Fluoxastrobin | 361377-29-9 | Sigma-Aldrich |
| Fungicide | Mitochondrial toxicants | Picoxystrobin | 117428-22-5 | Sigma-Aldrich |
| Fungicide | Mitochondrial toxicants | Pyraclostrobin | 175013-18-0 | Sigma-Aldrich |
| Fungicide | Mitochondrial toxicants | Trifloxystrobin | 141517-21-7 | Sigma-Aldrich |
| Fungicide | Mitochondrial toxicants | Hexachlorophene | 70-30-4 | Sigma-Aldrich |
| Herbicide | Redox cycler | Diquat dibromide monohydrate | 6385-62-2 | Sigma-Aldrich |
| Herbicide | Redox cycler | Paraquat dichloride hydrate | 75365-73-0 | Sigma-Aldrich |
| Industrial chemical | Baseline toxicants | 2-Phenylphenol | 90-43-7 | Sigma-Aldrich |
| Industrial chemical | Baseline toxicants | 3-Nitroaniline | 99-09-2 | Sigma-Aldrich |
| Industrial chemical | Baseline toxicants | 4-Chloro-3-methylphenol | 59-50-7 | Sigma-Aldrich |
| Industrial chemical | Baseline toxicants | 4-Pentylphenol | 14938-35-3 | Sigma-Aldrich |
| Industrial chemical | Baseline toxicants | 2-Allylphenol | 1745-81-9 | Sigma-Aldrich |
| Industrial chemical | Baseline toxicants | 2-Butoxyethanol | 111-76-2 | Sigma-Aldrich |
| Industrial chemical | EDC | Di(2-ethylhexyl) phthalate | 117-81-7 | Fluka |
| Industrial chemical | EDC | Bisphenol A | 80-05-7 | Sigma-Aldrich |
| Industrial chemical | EDC | 3,3',5,5'-Tetrabromobisphenol A | 79-94-7 | Sigma-Aldrich |
| Combustion by-product (PAH) | | Benz[a]anthracene | 56-55-3 | Sigma-Aldrich |
| Combustion by-product (PAH) | | Benzo[a]pyrene | 50-32-8 | Sigma-Aldrich |
| Combustion by-product (PAH) | | Anthracene | 120-12-7 | Sigma-Aldrich |
| Combustion by-product (PAH) | | Pyrene | 129-00-0 | abcr |
| Flame retardant (BDE) | | BDE-47 | 5436-43-1 | CPA Chem |
| Flame retardant (BDE) | | BDE-99 | 60348-60-9 | Sigma-Aldrich |
| Flame retardant (BDE) | | BDE-153 | 68631-49-2 | CPA Chem |
| Industrial chemical (PCB) | | PCB-2 | 2051-61-8 | Dr. Ehrenstorfer |
| Industrial chemical (PCB) |  | PCB-11 | 2050-67-1 | Dr. Ehrenstorfer |
| Industrial chemical (PCB) |  | PCB-28 | 7012-37-5 | Dr. Ehrenstorfer |
| Industrial chemical (PCB) |  | PCB-52 | 35693-99-3 | Fluka |
| Industrial chemical (PCB) |  | PCB 53 | 41464-41-9 | Dr. Ehrenstorfer |

Endocrine disrupting chemicals (EDC); Polycyclic aromatic hydrocarbons (PAH); Brominated diphenyl ethers (BDE); Polychlorinated biphenyls (PCB).

## **Table S2. IC_10,baseline_ predicted based on liposome-water partition constants (*K*_lip/w_) with baseline toxicity QSAR (Eq. S1; Lee et al. (2021)) and the highest tested concentrations of inactive chemicals both on cell viability and neurite outgrowth.**

| Chemical name | log*K*_lip/w_ | Source | log(1/IC_10,baseline_(M)) | Highest tested concentration (M) |
| --- | --- | --- | --- | --- |
| Acrinathrin | 6.4 | *K*_ow_ QSAR^a^ | 5.35 | > IC_10,baseline_ |
| α-Cypermethrin | 6.0 | LSERD^b^ | 5.24 | > IC_10,baseline_ |
| Cyfluthrin | 6.1 | *K*_ow_ QSAR | 5.26 | > IC_10,baseline_ |
| Cyhalothrin | 7.0 | *K*_ow_ QSAR | 5.52 | > IC_10,baseline_ |
| Deltamethrin | 6.4 | *K*_ow_ QSAR | 5.34 | > IC_10,baseline_ |
| Esfenvalerate | 7.7 | LSERD | 5.70 | > IC_10,baseline_ |
| Permethrin | 6.9 | LSERD | 5.50 | > IC_10,baseline_ |
| β-Endosulfan | 4.4 | LSERD | 4.58 | > IC_10,baseline_ |
| Endosulfan sulfate | 3.5 | LSERD | 4.12 | 5.33^.^10^-5^ |
| Benz[a]anthracene | 6.0 | LSERD | 5.23 | > IC_10,baseline_ |
| Benzo[a]pyrene | 7.0 | Endo et al. (2011) | 5.52 | > IC_10,baseline_ |
| Anthracene | 4.8 | LSERD | 4.76 | 1.20^.^10^-5^ |
| Pyrene | 5.4 | LSERD | 4.98 | 2.39^.^10^-5^ |
| BDE-47 | 6.7 | Endo et al. (2013) | 5.44 | > IC_10,baseline_ |
| BDE-99 | 7.1 | Endo et al. (2013) | 5.55 | > IC_10,baseline_ |
| BDE-153 | 7.6 | Endo et al. (2013) | 5.69 | 8.67^.^10^-7^ |
| PCB-2 | 4.8 | LSERD | 4.75 | > IC_10,baseline_ |
| PCB-11 | 5.3 | LSERD | 4.95 | > IC_10,baseline_ |
| PCB-28 | 5.7 | Quinn et al. (2014) | 5.11 | > IC_10,baseline_ |
| PCB-52 | 5.9 | Quinn et al. (2014) | 5.19 | > IC_10,baseline_ |
| PCB 53 | 5.6 | LSERD | 5.09 | > IC_10,baseline_ |

^a^*K*_ow_ QSAR: QSAR equation using octanol-water partition constants (*K*_ow_) (Endo et al. 2011).

^b^LSERD: linear solvation energy relationships (Ulrich et al. 2017).

## **Fig. S1. Examples for image analysis in IncuCyte S3 live cell imaging system**

## Phase-contrast image (A) to measure neurite outgrowth using the developed mask (B) and fluorescence image (C) to measure cell viability using the developed mask (D).

**
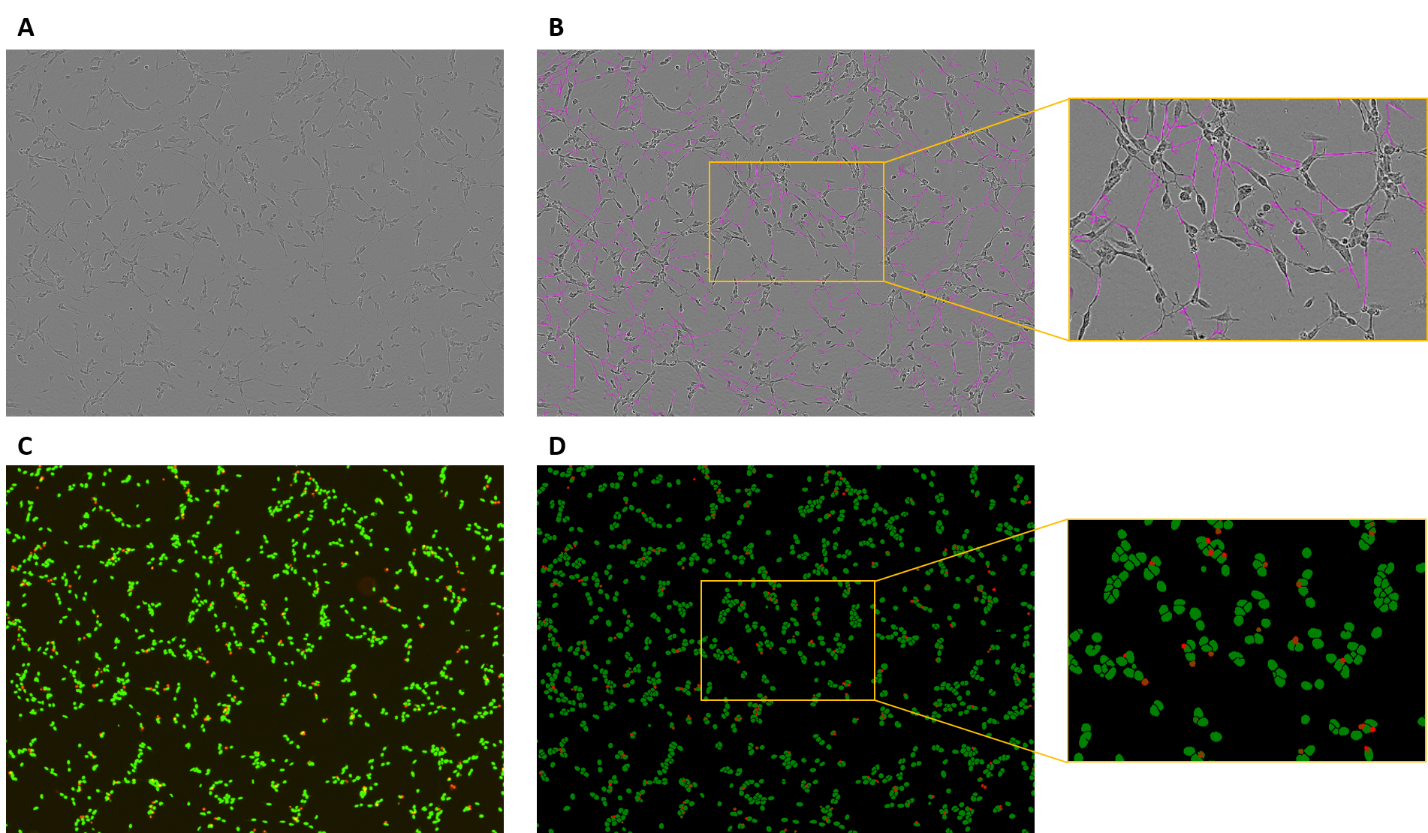
**

## **Fig. S2. Decision tree for selection of the model applied to fit concentration-response curve of (A) cell viability and (B) neurite length.**


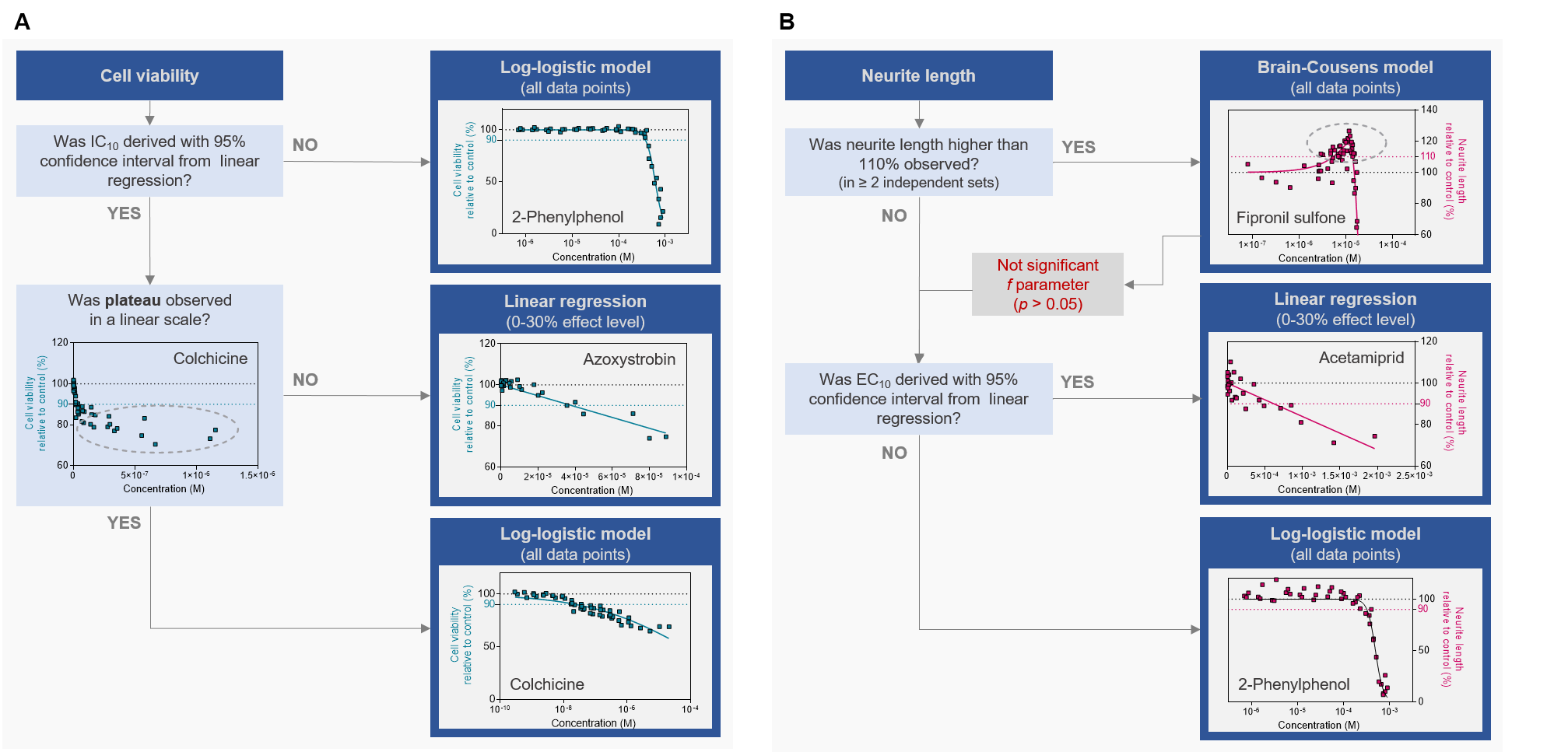


## **Text S1. Prediction of IC_10,baseline_ with a baseline toxicity prediction model**

A baseline toxicity prediction model was established specifically for differentiated SH-SY5Y cells to predict baseline toxicity (Lee et al. 2021). Nominal concentrations leading to 10% cytotoxicity by baseline toxicity (IC_10,baseline_) can be predicted from the liposome-water partition constants (*K*_lip/w_) with Eq. S1.

$\text{log(1/IC}_{\text{10,baseline}}\text{)=1.26+5.63×}\left( \text{1-}\text{e}^{\text{-0.202} {\text{log}\text{K}}_{\text{lip/w}}} \right)$ (S1)

Experimental *K*_lip/w_ were used preferentially if available, and for chemicals without experimental values, *K*_lip/w_ values were predicted based on linear solvation energy relationships (LSERD) (Ulrich et al. 2017) or QSAR equation using octanol-water partition constants (*K*_ow_). For anionic chemicals (hexachlorophene and TBBPA), the ionization-corrected distribution ratio *D*_lip/w_(pH 7.4) was used instead of *K*_lip/w_ considering speciation at the medium pH 7.4, however, high uncertainty is expected from these anionic chemicals as discussed in Lee et al. (2021).


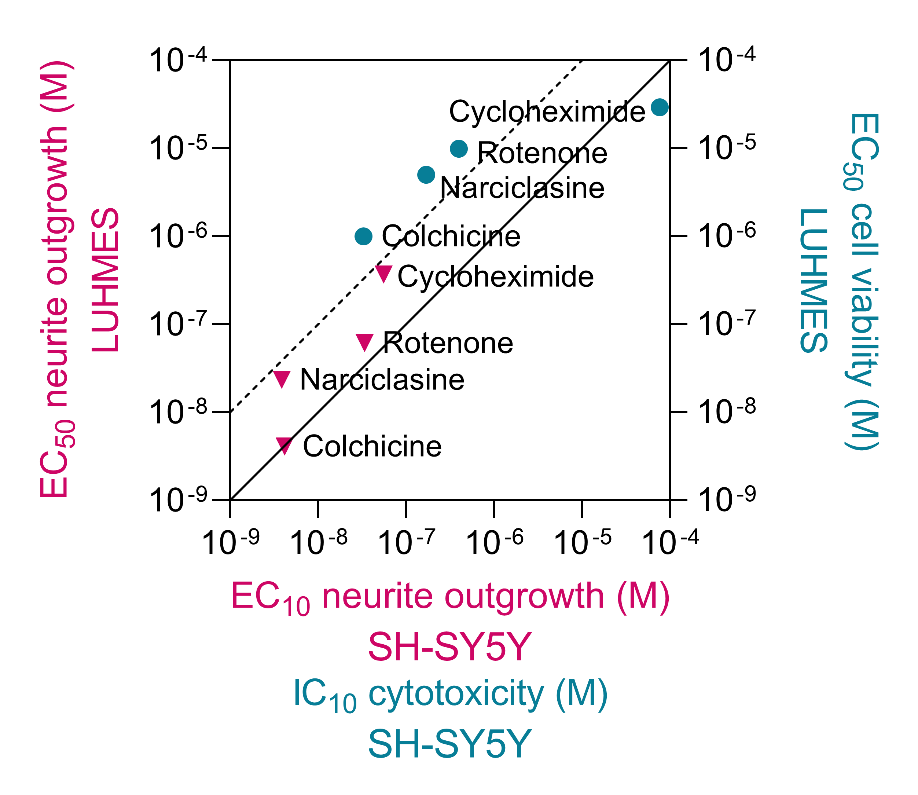


## **Fig. S3. Comparison of IC_10_ for cytotoxicity and EC_10_ for neurite outgrowth inhibition in SH-SY5Y cells compared well with EC_50_ for cytotoxicity and EC_50_ for neurite outgrowth inhibition observed in LUHMES cells (Krug et al. 2013).**

## **Table S3.** Best-fit model parameters for determination of effect concentrations for cytotoxicity (IC_10_) and neurite outgrowth inhibition (EC_10_)

| Group | Chemical  name | Cytotoxicity (IC_10_) | | | Neurite outgrowth inhibition (EC_10_) | | | | | | | | |
| --- | --- | --- | --- | --- | --- | --- | --- | --- | --- | --- | --- | --- | --- |
|  |  | Model^a^ | Slope | SE or CI^b^ | Model^a^ | Linear regression or  log-logistic model | | Brain-Cousens model^c^ | | | | | |
|  |  |  |  |  |  | Slope | SE or CI^b^ | b | SE | e | SE | f | SE |
| Endpoint-specific controls | Narciclasine | L | 59902105 | 5695445 | L | 2542188852 | 234409585 | - | - | - | - | - | - |
|  | Cycloheximide | LL | 0.57 | [0.52, 0.62] | L | 178759271 | 24652811 | - | - | - | - | - | - |
|  | Colchicine | LL | 0.28 | [0.24, 0.32] | L | 2365365472 | 280432630 | - | - | - | - | - | - |
|  | Rotenone | L | 25282416 | 1995536 | L | 295773729 | 37751992 | - | - | - | - | - | - |
| Baseline toxicants | 2-Butoxyethanol | L | 1154 | 78 | L | 2033 | 447 | - | - | - | - | - | - |
|  | 3-Nitroaniline | L | 12417 | 699 | L | 37999 | 5025 | - | - | - | - | - | - |
|  | 2-Allylphenol | L | 13627 | 668 | L | 22163 | 2219 | - | - | - | - | - | - |
|  | 4-Chloro-3-methylphenol | L | 29287 | 855 | L | 125514 | 15785 | - | - | - | - | - | - |
|  | 2-Phenylphenol | LL | 4.59 | [3.87, 5.47] | LL | 5.11 | [4.03, 6.67] | - | - | - | - | - | - |
|  | 4-Pentylphenol | LL | 13.05 | [9.44, 18.69] | L | 141966 | 13924 | - | - | - | - | - | - |
| AChE inhibitors | 3-Hydroxycarbofuran | L | 61587 | 4374 | L | 1672496 | 244987 | - | - | - | - | - | - |
|  | Carbaryl | L | 110372 | 7859 | L | 2058625 | 281433 | - | - | - | - | - | - |
|  | Diazoxon | L | 7899 | 936 | L | 37507 | 3648 | - | - | - | - | - | - |
|  | Paraoxon-ethyl | L | 20246 | 1611 | L | 95070 | 9806 | - | - | - | - | - | - |
|  | Chlorpyrifos-oxon | LL | 7.94 | [6.61, 9.49] | L | 300711 | 34640 | - | - | - | - | - | - |
| nAChR agonists | Thiamethoxam | Inactive | | | L | 25322 | 2436 | - | - | - | - | - | - |
|  | Imidacloprid | Inactive | | | L | 46999 | 5862 | - | - | - | - | - | - |
|  | Thiacloprid | Inactive | | | L | 142554 | 23951 | - | - | - | - | - | - |
|  | Acetamiprid | L | 6827 | 379 | L | 16086 | 1592 | - | - | - | - | - | - |
|  | Clothianidin | Inactive | | | L | 107628 | 12931 | - | - | - | - | - | - |
| GABA blockers | Fipronil | LL | 8.38 | [7.13, 9.78] | B | - | - | 6.37 | 0.76 | 52.98 | 0.97 | 0.31 | 0.09 |
|  | Fipronil sulfone | LL | 7.65 | [6.40, 9.10] | B | - | - | 8.34 | 0.79 | 17.44 | 0.22 | 1.89 | 0.24 |
|  | a-Endosulfan | LL | 8.32 | [6.38, 11.04] | B | - | - | 8.73 | 1.56 | 46.86 | 1.18 | 0.56 | 0.15 |
|  | Dieldrin | L | 241958 | 17319 | B | - | - | 10.66 | 2.69 | 63.09 | 1.00 | 0.21 | 0.07 |
| Sodium channel agonists | Bifenthrin | L | 929179 | 55857 | B | - | - | 2.12 | 0.21 | 22.30 | 2.27 | 2.97 | 0.74 |
|  | 4,4'-DDT | L | 884673 | 70958 | B | - | - | 7.03 | 0.99 | 14.32 | 0.32 | 1.52 | 0.40 |
| Mitochondrial toxicants | Azoxystrobin | L | 263555 | 14452 | L | 1346015 | 346991 | - | - | - | - | - | - |
|  | Picoxystrobin | Inactive | | | L | 920436 | 179084 | - | - | - | - | - | - |
|  | Fluoxastrobin | Inactive | | | L | 1513511 | 215472 | - | - | - | - | - | - |
|  | Pyraclostrobin | Inactive | | | L | 2142622 | 402564 | - | - | - | - | - | - |
|  | Trifloxystrobin | Inactive | | | L | 1086485 | 300785 | - | - | - | - | - | - |
|  | Hexachlorophene | L | 239426 | 20067 | B | - | - | 1.39 | 0.03 | 4.10 | 0.90 | 32.46 | 8.45 |
| Redox cyclers | Paraquat | LL | 0.32 | [0.28, 0.36] | L | 236088 | 34182 | - | - | - | - | - | - |
|  | Diquat | LL | 0.64 | [0.58, 0.70] | L | 4391431 | 494832 | - | - | - | - | - | - |
| Endocrine disruptors | Bisphenol A | L | 49166 | 6539 | L | 195318 | 25429 | - | - | - | - | - | - |
|  | 3,3′,5,5′-Tetra- bromobisphenol A | L | 92826 | 6073 | B | - | - | 7.96 | 1.07 | 197.25 | 3.60 | 0.06 | 0.02 |
|  | Di(2-ethylhexyl) phthalate | LL | 2.26 | [1.83, 2.80] | LL | 2.52 | [1.97, 3.23] | - | - | - | - | - | - |

^a^L: linear regression; LL: log-logistic model; B: Brain-Cousens model

^b^Standard error (SE) for slope in linear regression; 95% confidence interval (CI) for slope in log-logistic model

^c^Model parameters for Brain-Cousens model (Eq. 6); c is fixed at 0 (min) and d is fixed at 100 (max).

**(A) Endpoint-specific controls**

Narciclasine

Cycloheximide

Colchicine

Rotenone

## **Fig. S4. Concentration-response curves for tested chemicals in this study**

## (A) Endpoint-specific controls, (B) baseline toxicants, (C) AChE inhibitors, (D) nAChR agonists, (E) GABA receptor blockers, (F) sodium channel agonists, (G) mitochondrial toxicants, (H) redox cyclers, and (I) endocrine disruptors. Only the filled red data points were used for derivation of the linear concentration response curves, the greyed data points were not included.

**(B) Baseline toxicants**

2-Butoxyethanol

3-Nitroaniline

2-Allylphenol

4-Chloro-3-methylphenol

## **Fig. S4. Continued. Concentration-response curves for tested chemicals in this study**

## (A) Endpoint-specific controls, (B) baseline toxicants, (C) AChE inhibitors, (D) nAChR agonists, (E) GABA receptor blockers, (F) sodium channel agonists, (G) mitochondrial toxicants, (H) redox cyclers, and (I) endocrine disruptors.

2-Phenylphenol

4-Pentylphenol

**(C) AChE inhibitors**

3-Hydroxycarbofuran

Carbaryl

## **Fig. S4. Continued. Concentration-response curves for tested chemicals in this study**

## (A) Endpoint-specific controls, (B) baseline toxicants, (C) AChE inhibitors, (D) nAChR agonists, (E) GABA receptor blockers, (F) sodium channel agonists, (G) mitochondrial toxicants, (H) redox cyclers, and (I) endocrine disruptors.

Diazoxon

Paraoxon-ethyl

Chlorpyrifos-oxon

**(D) nAChR agonists**

Thiamethoxam

## **Fig. S4. Continued. Concentration-response curves for tested chemicals in this study**

## (A) Endpoint-specific controls, (B) baseline toxicants, (C) AChE inhibitors, (D) nAChR agonists, (E) GABA receptor blockers, (F) sodium channel agonists, (G) mitochondrial toxicants, (H) redox cyclers, and (I) endocrine disruptors.

Imidacloprid

Thiacloprid

Acetamiprid

Clothianidin

## **Fig. S4. Continued. Concentration-response curves for tested chemicals in this study**

## (A) Endpoint-specific controls, (B) baseline toxicants, (C) AChE inhibitors, (D) nAChR agonists, (E) GABA receptor blockers, (F) sodium channel agonists, (G) mitochondrial toxicants, (H) redox cyclers, and (I) endocrine disruptors.

**(E) GABA receptor blockers**

Fipronil

Fipronil sulfone

α-Endosulfan

Dieldrin

## **Fig. S4. Continued. Concentration-response curves for tested chemicals in this study**

## (A) Endpoint-specific controls, (B) baseline toxicants, (C) AChE inhibitors, (D) nAChR agonists, (E) GABA receptor blockers, (F) sodium channel agonists, (G) mitochondrial toxicants, (H) redox cyclers, and (I) endocrine disruptors.

**(F) Sodium channel agonists**

Bifenthrin

4,4'-DDT

**(G) Mitochondrial toxicants**

Azoxystrobin

Picoxystrobin

## **Fig. S4. Continued. Concentration-response curves for tested chemicals in this study**

## (A) Endpoint-specific controls, (B) baseline toxicants, (C) AChE inhibitors, (D) nAChR agonists, (E) GABA receptor blockers, (F) sodium channel agonists, (G) mitochondrial toxicants, (H) redox cyclers, and (I) endocrine disruptors.

Fluoxastrobin

Pyraclostrobin

Trifloxystrobin

Hexachlorophene

## **Fig. S4. Continued. Concentration-response curves for tested chemicals in this study**

## (A) Endpoint-specific controls, (B) baseline toxicants, (C) AChE inhibitors, (D) nAChR agonists, (E) GABA receptor blockers, (F) sodium channel agonists, (G) mitochondrial toxicants, (H) redox cyclers, and (I) endocrine disruptors.

**(H) Redox cyclers**

Paraquat

Diquat

**(I) Endocrine disruptors**

Bisphenol A

3,3′,5,5′-Tetrabromobisphenol A

## **Fig. S4. Continued. Concentration-response curves for tested chemicals in this study**

## (A) Endpoint-specific controls, (B) baseline toxicants, (C) AChE inhibitors, (D) nAChR agonists, (E) GABA receptor blockers, (F) sodium channel agonists, (G) mitochondrial toxicants, (H) redox cyclers, and (I) endocrine disruptors.

Di(2-ethylhexyl) phthalate

## **Fig. S4. Continued. Concentration-response curves for tested chemicals in this study**

## (A) Endpoint-specific controls, (B) baseline toxicants, (C) AChE inhibitors, (D) nAChR agonists, (E) GABA receptor blockers, (F) sodium channel agonists, (G) mitochondrial toxicants, (H) redox cyclers, and (I) endocrine disruptors.

## **Text S2. Determination of threshold to define neurite-specific effects.**

We derived limit of detection (LOD) for SR_cytotoxicity_ to determine threshold for neurite-specific effects. We assumed that known baseline toxicants can provide baseline level of SR_cytotoxicity_ considering their nonspecific effects on neurite outgrowth. SR_cytotoxicity_ (Eq. S2) and its standard error (SE; Eq. S3) applied for this calculation were from four known baseline toxicants whose SE of both IC_10_ and EC_10_ were available (2-allylphenol, 2-butoxyethanol, 3-nitroaniline, 4-chloro-3-methylphenol; Table S4). SR_cytotoxicity_ and SE from individual chemicals were substituted into Eq. 4, which resulted in LOD ranging from 1.9 to 4.6 (Table S4) with a mean LOD of 3.1.

$\text{SR}_{\text{cytotoxicity}}\text{=}\frac{\text{IC}_{\text{10}}}{\text{EC}_{\text{10}}}$ (S2)

$\text{σ}\text{SR}_{\text{cytotoxicity}}\text{=}\sqrt{\left( \frac{\text{∂}\text{SR}_{\text{cytotoxicity}}}{\text{∂}\text{IC}_{\text{10}}} \right)^{\text{2}}\text{∙}\left( \text{σ}\text{IC}_{\text{10}} \right)^{\text{2}}\text{+}\left( \frac{\text{∂}\text{SR}_{\text{cytotoxicity}}}{\text{∂}\text{EC}_{\text{10}}} \right)^{\text{2}}\text{∙}\left( \text{σ}\text{EC}_{\text{10}} \right)^{\text{2}}}$

$\text{=}\sqrt{\left( \frac{\text{1}}{\text{EC}_{\text{10}}} \right)^{\text{2}}\text{∙}\left( \text{σI}\text{C}_{\text{10}} \right)^{\text{2}}\text{+}\left( \frac{\text{I}\text{C}_{\text{10}}}{{\text{EC}_{\text{10}}}^{\text{2}}} \right)^{\text{2}}\text{∙}\left( \text{σ}\text{EC}_{\text{10}} \right)^{\text{2}}}$ $\text{=}\sqrt{\frac{\text{1}}{{\text{EC}_{\text{10}}}^{\text{2}}}\text{∙}\left( \text{σI}\text{C}_{\text{10}} \right)^{\text{2}}\text{+}\frac{{\text{IC}_{\text{10}}}^{\text{2}}}{{\text{EC}_{\text{10}}}^{\text{4}}}\text{∙}\left( \text{σ}\text{EC}_{\text{10}} \right)^{\text{2}}}$ (S3)

$\text{Limit of detection }\left( \text{LOD} \right)\text{ = }\text{SR}_{\text{cytotoxicity}}\text{+3×σ}\text{SR}_{\text{cytotoxicity}}$ (S4)

## **Table S4. IC_10_, EC_10_, SR_cytotoxicity_, and their standard error (SE) of known baseline toxicants used for determining limit of detection (LOD).**

| Chemical name | Cytotoxicity | |  | Neurite outgrowth inhibition | |  | Neurite-specificity ratio | | |
| --- | --- | --- | --- | --- | --- | --- | --- | --- | --- |
|  | IC_10_ (M) | SE |  | EC_10_ (M) | SE |  | SR_cytotoxicity_ | SE | LOD |
| 2-Allylphenol | 7.3^.^10^-4^ | 3.6^.^10^-5^ |  | 4.5^.^10^-4^ | 4.5^.^10^-5^ |  | 1.6 | 0.1 | 1.9 |
| 2-Butoxyethanol | 8.7^.^10^-3^ | 5.9^.^10^-4^ |  | 4.9^.^10^-3^ | 1.1^.^10^-3^ |  | 1.8 | 0.1 | 2.1 |
| 3-Nitroaniline | 8.1^.^10^-4^ | 4.5^.^10^-5^ |  | 2.6^.^10^-4^ | 3.5^.^10^-5^ |  | 3.1 | 0.2 | 3.7 |
| 4-Chloro-3-methylphenol | 3.4^.^10^-4^ | 1.0^.^10^-5^ |  | 8.0^.^10^-5^ | 1.0^.^10^-5^ |  | 4.3 | 0.1 | 4.6 |

Previously, Krug et al. (2013) defined a threshold of 4 to discriminate neurite-specific chemicals considering average + 3 standard deviation of the 50% effect concentration ratios for cytotoxicity and neurite outgrowth inhibition from unspecific chemicals (chemicals without MOAs relevant to neurite outgrowth). Considering that our LOD are very close to the threshold defined by Krug et al. (2013) but were determined from a limited number of chemicals, we adopted the threshold of 4 from Krug et al. (2013).

## **Text S3. Evaluation of endpoint-specific controls for stimulating effects on neurite outgrowth.**

Two endpoint-specific controls for stimulating neurite outgrowth, HA-1077 dihydrochloride and Y-27632 dihydrochloride (Aschner et al. 2017), were tested in SH-SY5Y cells as described in Materials and Methods. These chemicals were tested only once as we only aimed to confirm capacity of our assay to capture stimulating effects in a qualitative way and it should be noted that the effect concentrations (Table S5) derived for these two controls are highly uncertain (no repeat test) and extrapolated. Additionally, although the neurites shrunk and got thinner at high concentration, they were still quantified as intact neurites where most of the cells were dead already (Fig. S5).

## **Table S5. Stimulating effects of endpoint-specific controls on neurite outgrowth analyzed in Brain-Cousens model.**

| Chemicals | Stimulating effects | | EC_10_ (M) | | Maximum  neurite length (%) |
| --- | --- | --- | --- | --- | --- |
|  | *f* | *p*-value | inhibition | Stimulation^a^ |  |
| HA-1077 | 44.9 | 0.0008 | - | 3.15^.^10^-7^ | 163.5 |
| Y-27632 | 103.6 | 0.0369 | - | 1.64^.^10^-7^ | 186.2 |

^a^High uncertainty arises from extrapolation.

## **Fig. S5. Concentration-response curves of endpoint-specific controls for stimulating effects of neurite outgrowth of (A) HA-1077 and (B) Y-27632.**

## **Table S6. Stimulating effects on neurite outgrowth analyzed in Brain-Cousens model based on total neurite length or total neurite length divided by total cell counts (**p* < 0.05, ***p* < 0.01, ****p* < 0.001, *****p* < 0.0001).**

| Chemicals | Total neurite length | | |  | Total neurite length per total cells | |
| --- | --- | --- | --- | --- | --- | --- |
|  | *f* | *p*-value | EC_10_ (M) |  | *f* | *p*-value |
| TBBPA | 0.06 | 2.7^.^10^-3^** | NA |  | 0.17 | 1.4^.^10^-9^**** |
| Dieldrin | 0.21 | 3.6^.^10^-3^** | NA |  | 0.21 | 6.3^.^10^-3^** |
| Fipronil | 0.31 | 1.0^.^10^-3^** | NA |  | 0.41 | 1.1^.^10^-6^**** |
| α-Endosulfan | 0.56 | 1.9^.^10^-4^*** | 1.8^.^10^-5^ |  | 0.62 | 8.3^.^10^-6^**** |
| 4,4’-DDT | 1.52 | 2.8^.^10^-4^*** | 7.1^.^10^-6^ |  | 2.09 | 2.3^.^10^-5^**** |
| Fipronil sulfone | 1.89 | 3.4^.^10^-11^**** | 5.3^.^10^-6^ |  | 1.80 | 3.0^.^10^-10^**** |
| Bifenthrin | 2.97 | 2.5^.^10^-4^*** | 4.8^.^10^-6^ |  | 2.42 | 6.7^.^10^-3^** |
| Hexachlorophene | 32.46 | 4.1^.^10^-4^*** | 4.8^.^10^-7^ |  | 26.62 | 1.1^.^10^-4^*** |

NA: not available.

# References

Aschner M, Ceccatelli S, Daneshian M, et al. (2017) Reference Compounds for Alternative Test Methods to Indicate Developmental Neurotoxicity (DNT) Potential of Chemicals: Example Lists and Criteria for their Selection and Use. ALTEX 34(1):49-74 doi:10.14573/altex.1604201

Endo S, Escher BI, Goss KU (2011) Capacities of Membrane Lipids to Accumulate Neutral Organic Chemicals. Environ Sci Technol 45(14):5912-5921 doi:10.1021/es200855w

Endo S, Mewburn B, Escher BI (2013) Liposome and protein-water partitioning of polybrominated diphenyl ethers (PBDEs). Chemosphere 90:505-511 doi:10.1021/es200855w

Krug AK, Balmer NV, Matt F, Schonenberger F, Merhof D, Leist M (2013) Evaluation of a human neurite growth assay as specific screen for developmental neurotoxicants. Arch Toxicol 87(12):2215-31 doi:10.1007/s00204-013-1072-y

Lee J, Braun G, Henneberger L, et al. (2021) Critical Membrane Concentration and Mass-Balance Model to Identify Baseline Cytotoxicity of Hydrophobic and Ionizable Organic Chemicals in Mammalian Cell Lines. Chem Res Toxicol 34(9):2100-2109 doi:10.1021/acs.chemrestox.1c00182

Quinn CL, van der Heijden SA, Wania F, Jonker MTO (2014) Partitioning of Polychlorinated Biphenyls into Human Cells and Adipose Tissues: Evaluation of Octanol, Triolein, and Liposomes as Surrogates. Environ Sci Technol 48(10):5920-5928 doi:10.1021/es500090x

Ulrich N, Endo S, Brown TN, et al. (2017) UFZ-LSER database v 3.2.1, Helmholtz Centre for Environmental Research-UFZ, Leipzig, Germany [accessed on 05.02.2021]; <http://www.ufz.de/lserd>.
